# RkMsn4, a zinc finger transcription factor, regulates carotenoid and lipid metabolism to enhance tolerance to environmental stress in *Rhodosporidium kratochvilovae*

**DOI:** 10.1128/aem.00647-26

**Published:** 2026-09-23

**Authors:** Meixia He, Mingjing Yang, Xinyan Xiong, Yuxuan Gan, Jingwen Qiu, Yuan Chen, Qi Zhang

**Affiliations:** 1 Faculty of Life Science and Technology, Kunming University of Science and Technology 47910, Kunming, China; Chalmers tekniska hogskola AB, Gothenburg, Sweden

**Keywords:** *Rhodosporidium kratochvilovae*, environmental stress, transcription factor RkMsn4, carotenoids, unsaturated fatty acids

## Abstract

Red yeasts serve as indispensable microbial resources for the production of carotenoids and lipids; however, environmental stress factors often limit their performance during industrial fermentation. The zinc-finger transcription factors Msn2/Msn4 act as central regulators of stress responses in yeasts. However, researchers have not yet systematically elucidated the similarities and differences in the mechanisms underlying the regulatory effects of Msn2/Msn4 under different stress conditions. In this study, we compared the shared and stress-specific regulatory functions of RkMsn4 in *Rhodosporidium kratochvilovae* YM25235 under conditions of glucose starvation, 15 °C low temperature, and 4% NaCl salt stress. RkMsn4 contributed to the activity of the oxidative defense systems, including antioxidant enzymes, carotenoids, and glutathione, and the remodeling of membrane lipids, including phospholipids and unsaturated fatty acids (UFAs) across all three stress conditions. Additionally, RkMsn4 regulated stress-specific metabolic regulation by modulating branched-chain amino acid (BCAA) and aromatic amino acid (AAA) metabolism under glucose starvation conditions and promoting the utilization of exogenous sucrose and fructose under salt stress. By conducting weighted gene co-expression network analysis (WGCNA), we identified candidate genes potentially associated with carotenoid and lipid metabolism. These findings provide practical insights for improving tolerance to stress and metabolic productivity of red yeasts in industrial bioprocesses.

**IMPORTANCE:**

Red yeasts are important microorganisms for the production of carotenoids and lipids, but their performance is often limited by environmental stress. Understanding how red yeasts respond to environmental stress is crucial for advancing their application in industrial fermentation. This study reveals the stress response mechanisms mediated by the transcription factor RkMsn4 in *Rhodosporidium kratochvilovae* YM25235 under glucose starvation, low temperature, and salt stress, as well as its regulatory role in carotenoid and lipid metabolism. These findings provide valuable insights for enhancing the stress resistance and productivity of red yeasts in industrial applications.

## INTRODUCTION

Microorganisms are frequently challenged by dynamic and multifactorial environmental stress, such as nutrient limitation, temperature fluctuation, osmotic changes, and oxidative stress, in both natural environments and industrial bioprocesses (1–3). These adverse conditions not only severely constrain microbial growth and the robustness of the system but also directly affect the biosynthesis and efficiency of accumulation of various high-value secondary metabolites, including lipids, carotenoids, organic acids, and other metabolites with functional or industrial relevance (4–6). Transcriptional regulation is an efficient strategy by which cells respond to environmental stress (1, 7, 8). In several model yeasts and pathogenic yeasts, distinct stressors induce or repress a common set of genes defined as the core stress response or core environmental response (CER), which confers cross-protection against multiple adverse stresses (9, 10). Stress-specific transcriptional programs are superimposed on this general response and enable modular and tailored adaptations to distinct environmental challenges (11). This combination of general and specific regulation allows rapid and precise adaptation to stress, representing an evolutionarily efficient survival strategy (10). Several transcription factors (TFs) coordinate this network by regulating the transcription of large sets of downstream genes (9, 10). Among these TFs, Msn2 and Msn4 in *Saccharomyces cerevisiae* and *Candida glabrata* are important regulators of the expression of the CER gene (1, 8, 12, 13).

The zinc finger TFs Msn2 and Msn4 are important regulators activated under various adverse conditions, such as carbon starvation, heat shock, osmotic stress, oxidative stress, and high concentrations of ethanol in *S. cerevisiae* (12, 14–18). When exposed to stress, Msn2/Msn4 rapidly translocate to the nucleus and bind to stress response elements (STRE, 5′-AGGGG-3′ or 5′-CCCCT-3′) to regulate stress-responsive gene expression (12, 16). Specifically, Msn2/Msn4 induce genes encoding heat shock proteins (Hsps) to promote protein refolding (1, 14, 15), activate antioxidant systems such as catalase (CTT1) and superoxide dismutase (SOD1/2) to enhance the scavenging of reactive oxygen species (ROS) (12, 19), and modulate the synthesis of osmolytes, such as glycerol, trehalose, and proline, to maintain osmotic balance (16, 20–23). Moreover, Msn2/Msn4 regulate carbohydrate metabolism, the β-oxidation of fatty acids, and the biogenesis of peroxisomes, thereby increasing intracellular availability of acetyl-CoA and energy supply (1, 14, 24, 25). Although Msn2/Msn4-mediated stress responses are extensively elicited under different types of stress, substantial differences may exist in upstream signaling, Msn2/Msn4 activity and nucleo-cytoplasmic dynamics, and downstream transcriptional profiles (26–28). However, systematic comparative analyses of the mechanisms underlying the regulatory effects of Msn2/Msn4 under different stress conditions remain limited, and whether these mechanisms share common regulatory features or exhibit stress-specific differences needs to be determined.

Transcriptional regulation is often accompanied by the reorganization of the metabolic network and the accumulation of protective metabolites, including trehalose, carotenoids, glutathione (GSH), and various species of lipids in yeast (20, 29, 30). Among these, lipid metabolism plays an important role, as it directly affects membrane homeostasis, signal transduction, substance transport, and morphogenesis (31). Yeast cells increase tolerance to stress by modulating the composition and properties of lipids. Under hyperosmotic stress, an increase in phospholipid and sterol composition helps maintain the structure of the membrane bilayer in *Debaryomyces hansenii* (32). While heat stress promotes the accumulation of sterol to improve the stability of the membrane in *S. cerevisiae* (33). In contrast, when exposed to low temperature, high hydrostatic pressure, ethanol, or salt stress, yeasts typically increase fatty acid unsaturation to maintain membrane fluidity and improve oxidative defense capacity (29, 34–36). Lipid metabolism is closely associated with the biosynthesis of carotenoids. Carotenoids are potent natural antioxidants usually stored in lipid bodies, where they modulate the fluidity of the membrane and facilitate cold adaptation (37, 38), while also providing effective protection against oxidative stress under various adverse conditions (39, 40). Lipids and carotenoids act as key metabolic determinants of membrane stability and cellular robustness during stress adaptation. However, how the metabolism of lipids and carotenoids is regulated during stress responses, and whether Msn2 and Msn4 play a role in orchestrating these regulatory processes, needs to be assessed.

*Rhodosporidium* spp., efficient producers of carotenoids and lipids, are considered promising microbial cell factories (41, 42). *Rhodosporidium kratochvilovae* YM25235 is a strain that can simultaneously synthesize carotenoids and polyunsaturated fatty acids (PUFAs) (34, 43, 44). This strain was originally isolated from a high-altitude plateau lake characterized by low annual average temperature of 15°C, nutrient limitation, and fluctuating salinity (Chenghai Lake, Lijiang, Yunnan, China) (45, 46). The ecological environment and evolutionary history of YM25235 may have imparted it with unique stress response mechanisms and strong adaptability to the environment. PUFA and carotenoid biosynthesis in YM25235 is closely associated with adaptation to low temperature and glucose starvation (46–48). Therefore, the adaptive mechanisms of YM25235 in response to nutrient limitation, temperature variation, and salt (ionic) stress need to be elucidated, as these factors not only constitute key ecological pressures in the native habitat of YM25235 but also represent critical challenges encountered during industrial fermentation (30, 42).

In this study, we performed targeted deletion of *RkMsn4*, which encodes the Msn2/Msn4 homolog in *R. kratochvilovae* YM25235. We performed transcriptomic analysis and systematically elucidated RkMsn4-mediated regulatory networks under conditions of glucose starvation, low temperature, and salt stress. We also investigated the role of RkMsn4 in regulating carotenoid and lipid metabolism. This study provides the first comprehensive insights into how Msn4 integrates environmental stress signals with metabolic regulation in red yeast, offering new perspectives on stress adaptation and providing a theoretical basis for developing stress-tolerant, metabolite-rich bioengineering strains.

## RESULTS

### RkMsn4 affects stress adaptation of YM25235

**Fig 1:**
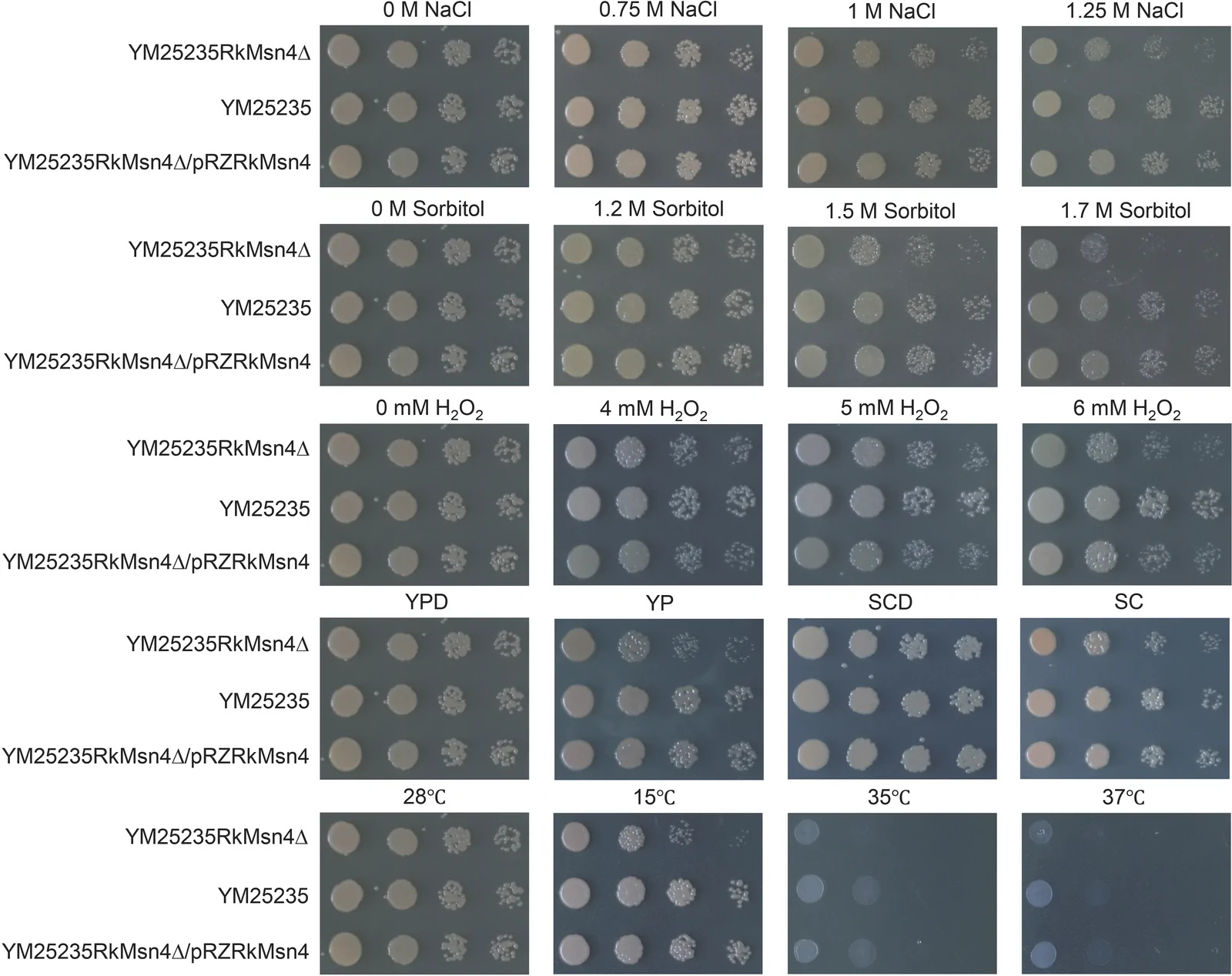
Spot assays under various types of stress. YPD, yeast extract-peptone-dextrose medium; YP, yeast extract-peptone medium without glucose; SCD, synthetic complete medium; SC, synthetic complete medium without glucose.

In preliminary experiments, we exposed the wild-type strain YM25235 and the *RkMsn4* deletion mutant to various environmental stresses, including salt, osmotic, low temperature, heat, glucose starvation, and oxidative stress, and assessed their effects on cell growth. Spot assays (Fig. 1) showed that on solid media supplemented with NaCl, sorbitol, or H_2_O_2_, the *RkMsn4* deletion mutant formed smaller and thinner colonies and exhibited markedly impaired growth compared with YM25235. The mutant also displayed obvious growth defects in glucose-free YP and SC media. At 15°C, the mutant grew poorly. Under heat stress at 35°C and 37°C, growth of both YM25235 and mutant strains was severely inhibited, and the mutant showed much weaker colony development. Complementation of *RkMsn4* partially restored the growth capacity of the mutant under the above stresses. However, the complemented strain still grew significantly worse than YM25235 in the presence of H_2_O_2_.

**Fig 2:**
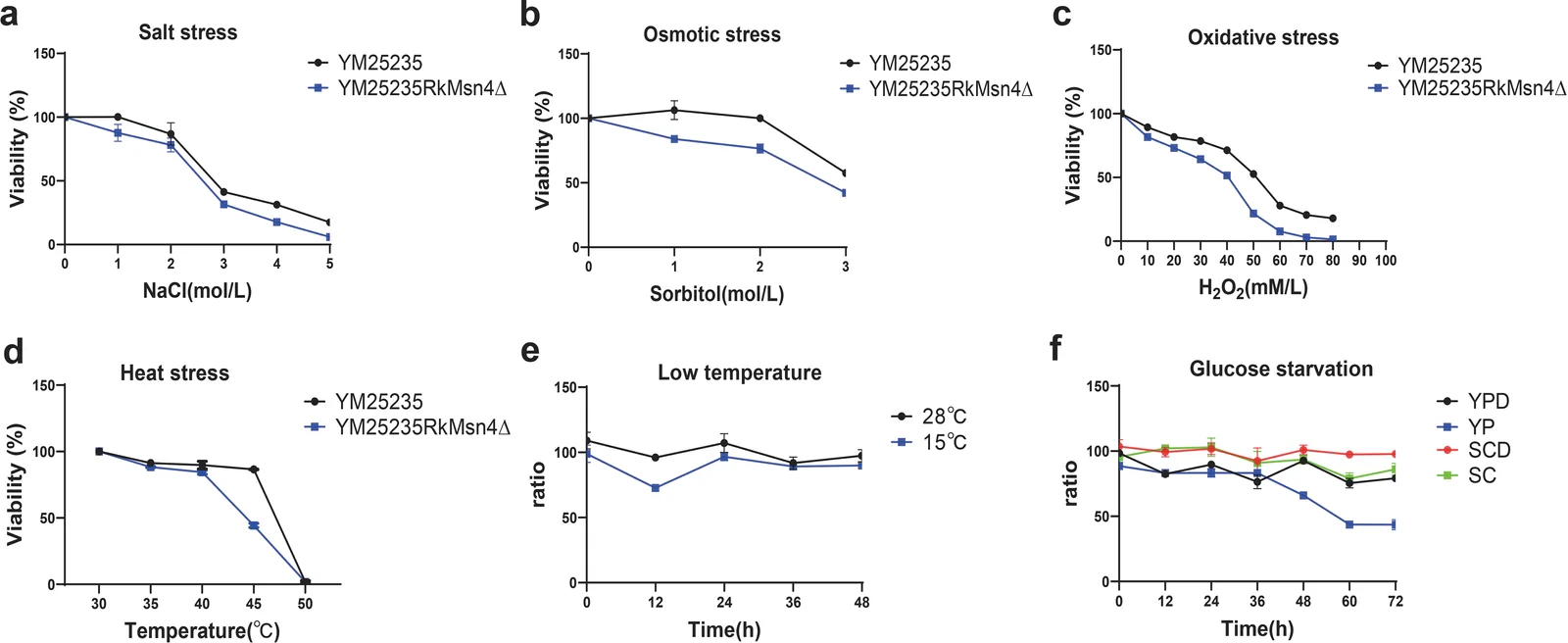
Survival rate analysis under various types of stress. Survival rates of the YM25235 and *RkMsn4* deletion mutant under (**a**) different concentrations of NaCl, (**b**) different concentrations of sorbitol, (**c**) different concentrations of H_2_O_2_, (**d**) heat shock, (**e**) incubation at 15°C, and (**f**) glucose-free medium. Panels e and f show the survival rate ratios of the *RkMsn4* deletion mutant to YM25235 under identical treatment conditions. YPD, yeast extract-peptone-dextrose medium; YP, yeast extract-peptone medium without glucose; SCD, synthetic complete medium; SC, synthetic complete medium without glucose.

Cell survival assays further confirmed the growth differences between YM25235 and the *RkMsn4* deletion mutant under stress conditions (Fig. 2). After short-term treatment with NaCl, sorbitol, H_2_O_2_, or heat shock, the survival rate of the *RkMsn4* deletion mutant declined sharply with increasing stress intensity, with a far greater reduction than that observed for YM25235. At 15°C, the mutant grew slowly within the first 24 h and then recovered gradually. In glucose-free YP and SC media, its survival rate was also significantly lower than that of YM25235. These results indicate that RkMsn4 is involved in the adaptation of strain YM25235 to multiple stresses. Combined with the natural habitat of strain YM25235 and our preliminary findings (46, 47), we selected glucose starvation (glucose-depleted YPD medium), low temperature at 15°C, and salt stress with 4% NaCl as typical stressors for subsequent systematic investigation. Notably, growth curve analyses in liquid cultures under these three stresses (Fig. S1) revealed no obvious differences in growth between the *RkMsn4* deletion mutant and YM25235.

### RkMsn4 regulates ROS levels, antioxidant enzyme gene expression, and carotenoid biosynthesis in YM25235

**Fig 3:**
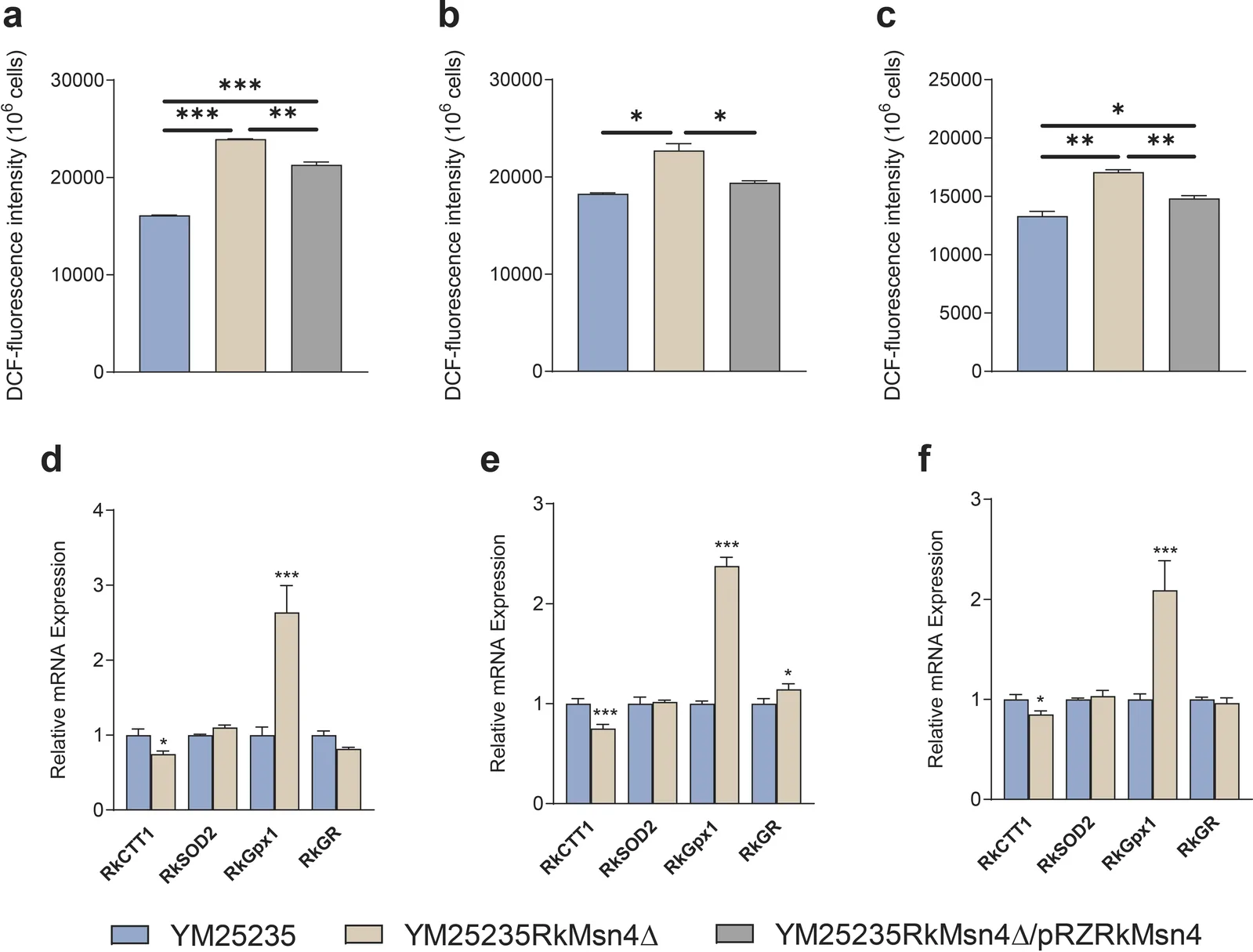
Analysis of ROS levels and transcript levels of antioxidant enzyme genes. (**a–c**) Intracellular ROS levels under glucose starvation, low temperature, and salt stress, respectively. (**d–f**) Transcript levels of antioxidant enzyme genes under glucose starvation, low temperature, and salt stress, respectively. *RkCTT1*, catalase gene; *RkSOD2*, superoxide dismutase gene; *RkGpx1*, glutathione peroxidase 1 gene; *RkGR*, glutathione reductase gene. *P < 0.05, **P < 0.01, and ***P < 0.001.

**Fig 4:**
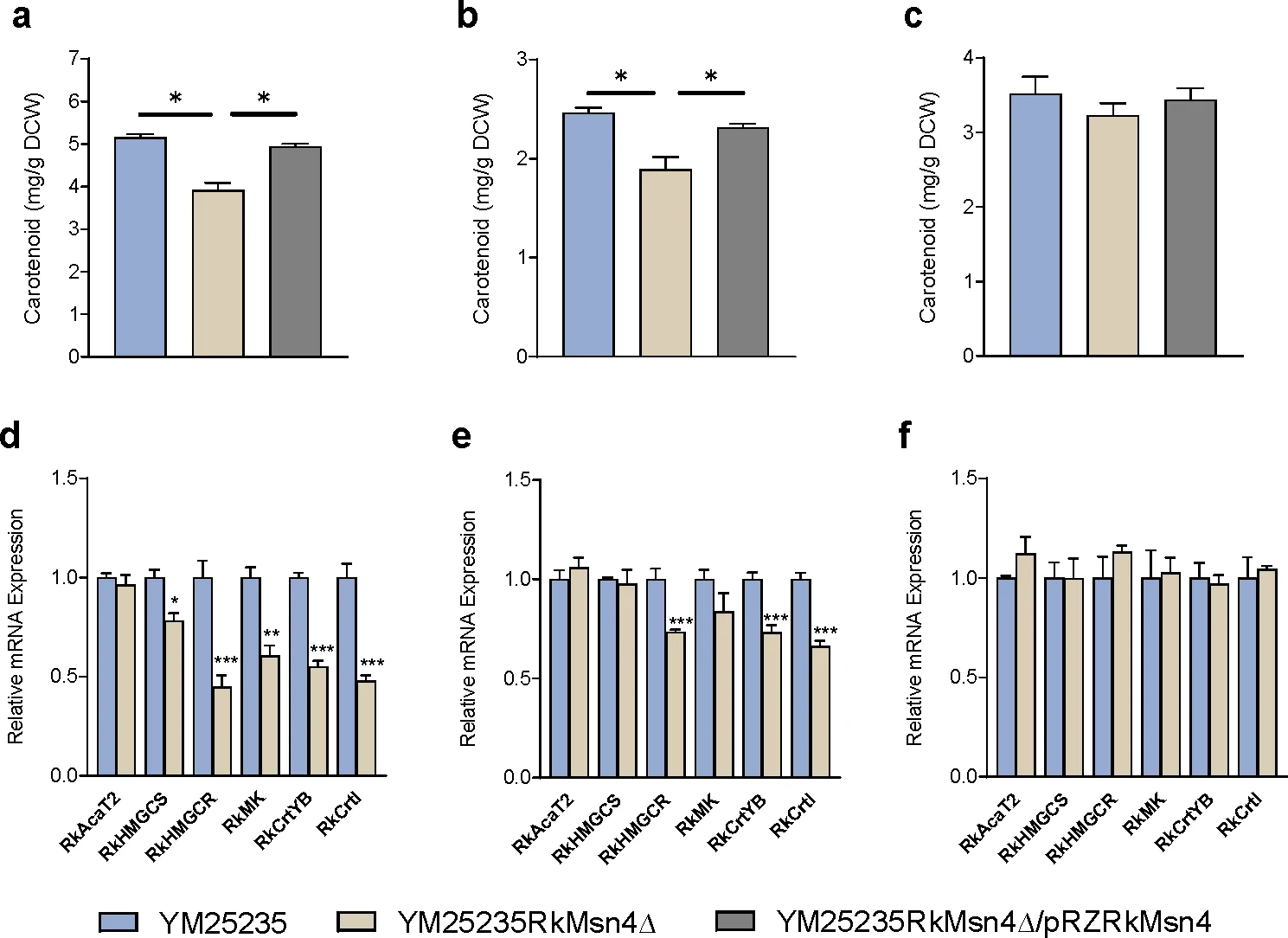
Analysis of total carotenoid contents and transcript levels of carotenoid biosynthesis-related genes. (**a–c**) Total carotenoid contents under glucose starvation, low temperature, and salt stress, respectively. (**d–f**) Transcript levels of carotenoid biosynthesis-related genes under glucose starvation, low temperature, and salt stress, respectively. DCW, dry cell weight; *RkAcaT2*, acetoacetyl-CoA thiolase gene; *RkHMGCS*, HMG-CoA synthase gene; *RkHMGCR*, HMG-CoA reductase gene; *RkMK*, mevalonate kinase gene; *RkCrtI*, phytoene dehydrogenase gene; *RkCrtYB*, phytoene synthase/lycopene cyclase gene. *P < 0.05, **P < 0.01, and ***P < 0.001.

Environmental stress often disrupts intracellular redox homeostasis and induces oxidative stress (49). Compared with wild-type strain YM25235, the *RkMsn4* deletion strain showed significantly higher levels of ROS under glucose starvation, low temperature, and salt stress, with increases of 48.48%, 24.36%, and 28.26%, respectively (Fig. 3a through c). Complementation of *RkMsn4* partially alleviated ROS accumulation but failed to restore ROS levels to those of the wild-type strain YM25235. Notably, the ROS content in the complemented strain remained markedly higher than that in YM25235 under glucose starvation and salt stress. Antioxidant enzymes serve as essential scavengers of intracellular ROS. Transcriptional analysis revealed that under the three stress conditions, the catalase gene *RkCTT1* exhibited a significantly lower expression level, whereas the glutathione peroxidase gene *RkGpx1* showed a dramatically higher expression level in the *RkMsn4* deletion mutant (Fig. 3d through f). Carotenoids are major antioxidants in YM25235. Relative to the wild-type strain YM25235, the *RkMsn4* deletion mutant showed 24.15% and 23.33% reductions in carotenoid content under glucose starvation and low temperature, respectively (Fig. 4a through c). A slight decrease of 8.21% was also observed under salt stress, which was not statistically significant (Fig. 4a through c). Transcriptional levels of key genes involved in the MVA pathway and carotenoid biosynthesis (*RkHMGCS*, *RkHMGCR*, *RkMK*, *RkCrtYB,* and *RkCrtI*) showed significantly lower expression under glucose starvation and low temperature (Fig. 4d through f). To further verify the regulatory effect of RkMsn4 on the expression of these genes, we performed promoter sequence analysis. The results demonstrated that the promoters of most genes related to antioxidant enzymes and carotenoid biosynthesis contain the STRE motifs, the binding site of transcription factors Msn2/Msn4 (Table S1). Collectively, these findings indicate that RkMsn4 plays a vital role in maintaining intracellular redox homeostasis in YM25235.

### RkMsn4 affects lipid accumulation and fatty acid composition in YM25235

**Fig 5:**
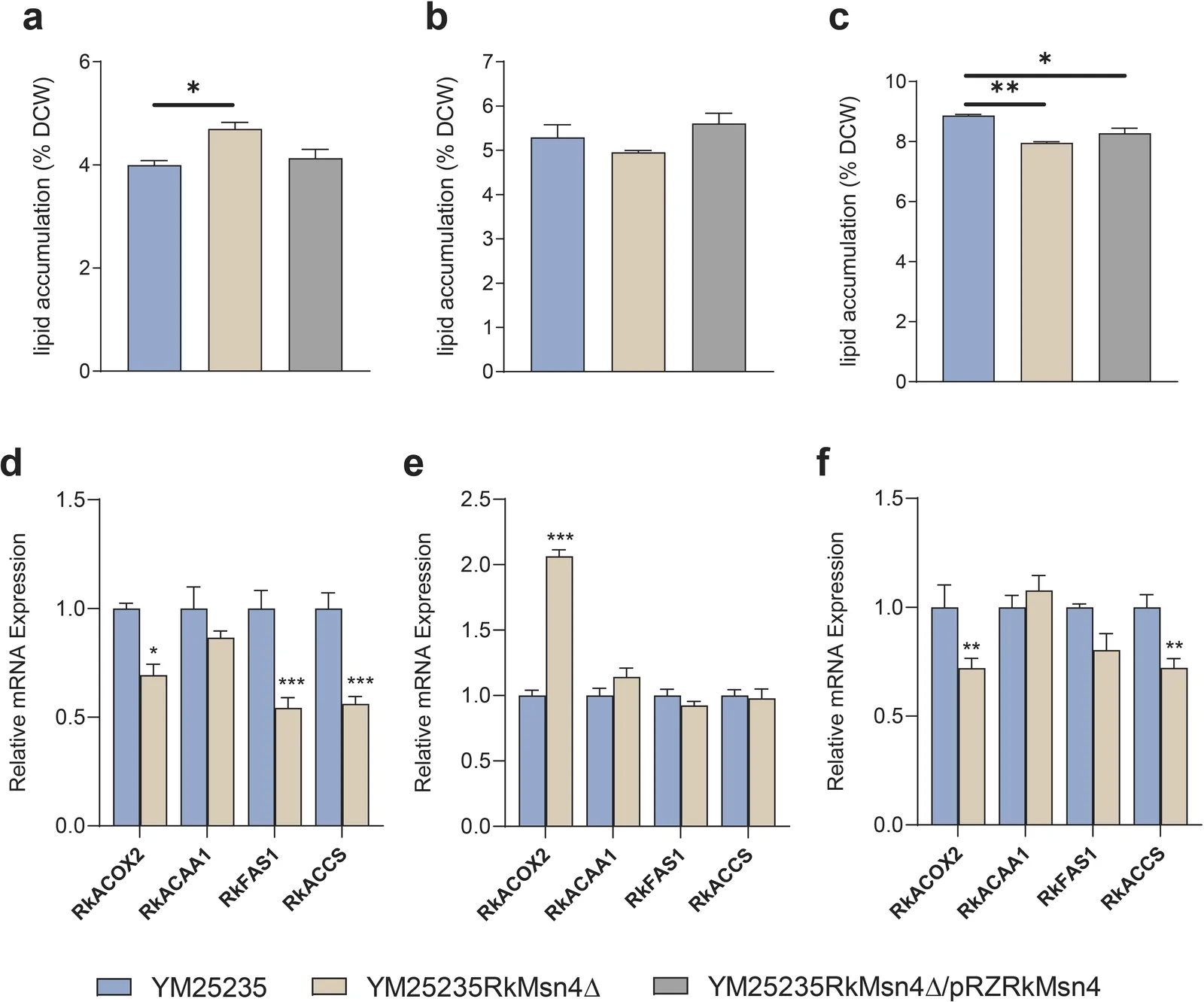
Analysis of total lipid contents and transcript levels of lipid metabolism-related genes. (**a–c**) Total lipid contents under glucose starvation, low temperature, and salt stress, respectively. (**d–f**) Transcript levels of lipid metabolism-related genes under glucose starvation, low temperature, and salt stress, respectively. DCW, dry cell weight; *RkACOX2*, fatty acyl-CoA oxidase gene; *RkACAA1*, 3-oxoacyl-CoA thiolase gene; *RkFAS1*, fatty acid synthase gene; *RkACCS*, acetyl-CoA carboxylase gene. *P < 0.05, **P < 0.01, and ***P < 0.001.

Previous studies have demonstrated that Msn2/Msn4 regulates fatty acid β-oxidation (24, 25). As shown in Fig. 5a through c, deletion of *RkMsn4* altered lipid accumulation relative to the wild-type strain YM25235. Under glucose starvation, lipid content increased by 17.58%. A 6.37% reduction in lipid accumulation was observed under low temperature, which was not statistically significant, while salt stress caused a significant decrease of 10.31%. Complementation of *RkMsn4* partially restored lipid metabolism in the mutant strain. Transcriptional analysis revealed that the expression of genes associated with fatty acid β-oxidation (*RkACOX2* and *RkACAA1*) and fatty acid synthesis (*RkFAS1* and *RkACCS*) were significantly lower under glucose starvation and salt stress (Fig. 5d through f). By contrast, *RkACOX2* exhibited a markedly higher expression level at low temperature. Promoter sequence analysis further identified the presence of STRE motifs in the promoter regions of *RkACOX2*, *RkACAA1,* and *RkFAS1* (Table S1). These results indicate that RkMsn4 modulates lipid metabolism in response to environmental stresses, and its regulatory effects vary with stress types.

Furthermore, fatty acid composition analysis (Table 1) revealed that relative to the wild-type strain YM25235, the *RkMsn4* deletion mutant exhibited increased levels of palmitic acid (C16:0) and stearic acid (C18:0), accompanied by reduced contents of palmitoleic acid (C16:1) and oleic acid (C18:1) under glucose starvation. Under low temperature, the content of palmitoleic acid (C16:1) decreased. When under salt stress, the levels of palmitic acid (C16:0), palmitoleic acid (C16:1), oleic acid (C18:1), and linoleic acid (C18:2) were all lowered. These results demonstrate that RkMsn4 modulates the biosynthesis of unsaturated fatty acids, especially monounsaturated fatty acids, in YM25235 under stress conditions.

**TABLE 1:** Analysis of the composition of fatty acids^*a*^

Stress condition	Strain	Total fatty acids (mg/g)	Fatty acid composition (mg/g)					
			C16:0	C16:1	C18:0	C18:1 OA	C18:2 LA	C18:3 ALA
Glucose starvation	YM25235	51.50 ± 0.34	8.94 ± 0.23	1.53 ± 0.01	2.23 ± 0.19	28.13 ± 0.12	9.51 ± 0.02	1.16 ± 0.03
	YM25235RkMsn4∆	51.73 ± 0.95	9.79 ± 0.22*	1.11 ± 0.01*	3.42 ± 0.02*	26.16 ± 0.00*	9.87 ± 0.62	1.37 ± 0.12
Low temperature	YM25235	44.86 ± 0.43	5.66 ± 1.59	0.53 ± 0.01	2.20 ± 1.15	13.20 ± 0.43	17.35 ± 0.28	2.91 ± 0.04
	YM25235RkMsn4∆	43.85 ± 0.18	7.06 ± 0.15	0.49 ± 0.01*	3.34 ± 0.22	13.14 ± 0.96	17.56 ± 0.06	3.27 ± 0.23
Salt stress	YM25235	60.05 ± 0.10	10.05 ± 0.11	1.34 ± 0.02	2.86 ± 0.12	38.78 ± 0.63	6.19 ± 0.28	0.83 ± 0.05
	YM25235RkMsn4∆	56.06 ± 0.02*	9.11 ± 0.01**	1.04 ± 0.00*	3.00 ± 0.00	36.62 ± 0.01**	5.49 ± 0.01*	0.80 ± 0.00

^*a*^ *P < 0.05, **P < 0.01.

### Transcriptome data quality assessment and identification of DEGs

To evaluate the effects of RkMsn4 on gene expression under glucose starvation, low temperature, and salt stress, we performed transcriptome sequencing. A total of 118.28 Gb of high-quality clean data were generated from 18 samples. Each sample yielded more than 5.90 Gb of clean data, with the Q30 ratio exceeding 94.40% (Table S2). The clean reads were mapped to the reference genome of *R. kratochvilovae* YM25235, and the mapping rates ranged from 76.01% to 85.38% (Table S2), demonstrating the high quality of the sequencing data.

**Fig 6:**
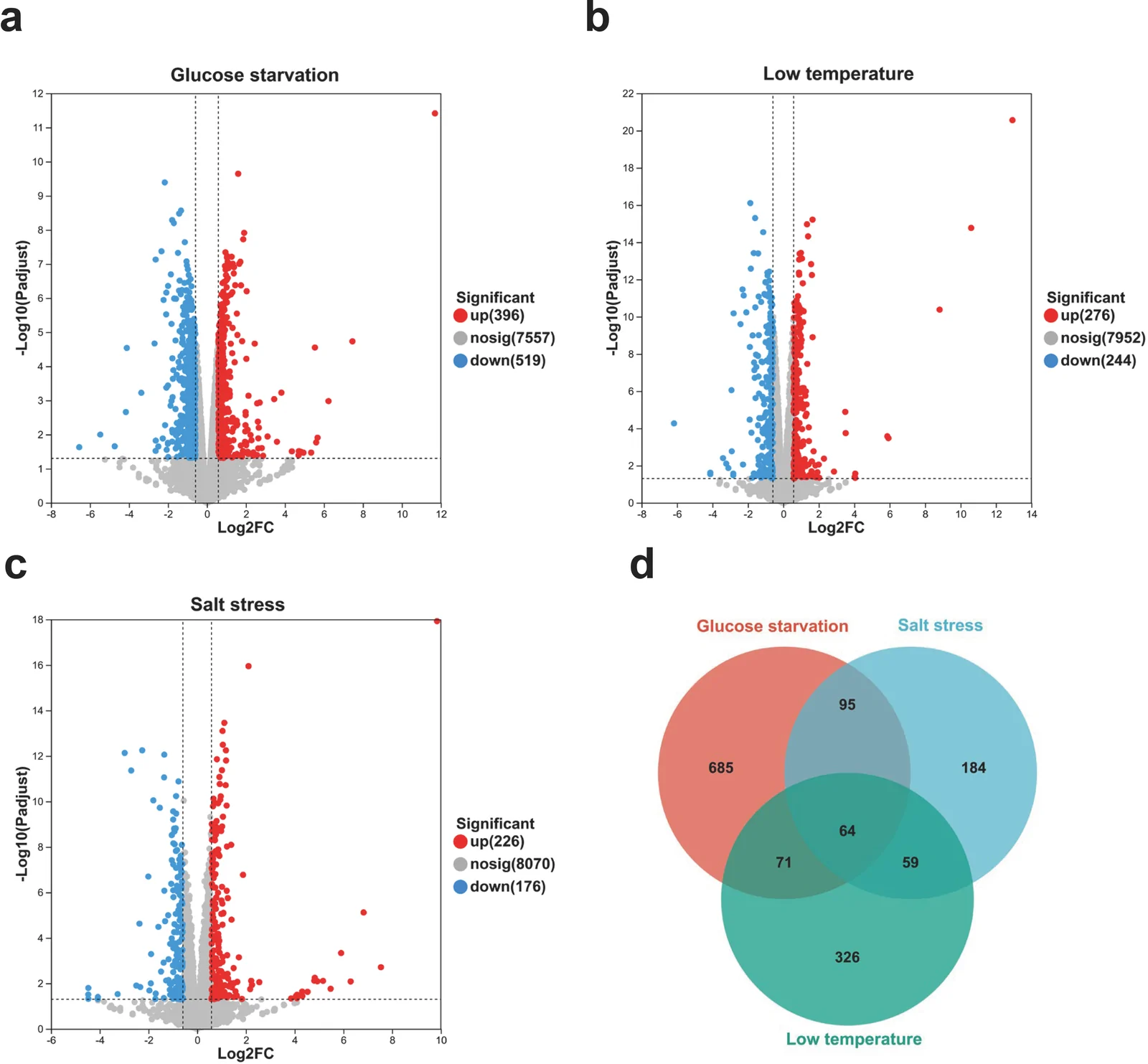
Identification of DEGs in the *RkMsn4* mutant relative to wild-type strain YM25235. Volcano plots show the DEGs under (**a**) glucose starvation, (**b**) low temperature, and (**c**) salt stress. (**d**) Venn analysis of the identified DEGs.

Differentially expressed genes (DEGs) were identified between the wild-type strain YM25235 and the *RkMsn4* deletion mutant under each stress condition. A total of 915 DEGs were detected under glucose starvation, including 396 genes with higher expression levels and 519 genes with lower expression levels (Fig. 6a and Table S3). Under low temperature, 520 DEGs were identified, with 276 genes showing higher expression levels and 244 genes showing lower expression levels (Fig. 6b and Table S3). A total of 402 DEGs were obtained under salt stress, comprising 226 genes with higher expression levels and 176 genes with lower expression levels (Fig. 6c and Table S3). Notably, 64 genes were differentially expressed across all three stress treatments. In addition, 685, 326, and 184 DEGs were specifically responsive to glucose starvation, low temperature, and salt stress, respectively (Fig. 6d). As validated by qRT-PCR (Fig. S2), the expression patterns of genes involved in carotenoid biosynthesis, lipid metabolism, and antioxidant enzyme were largely consistent with the RNA-Seq results, which confirmed the reliability and reproducibility of the transcriptome data.

### Integrated metabolic regulatory networks mediated by RkMsn4 under three types of stress

**Fig 7:**
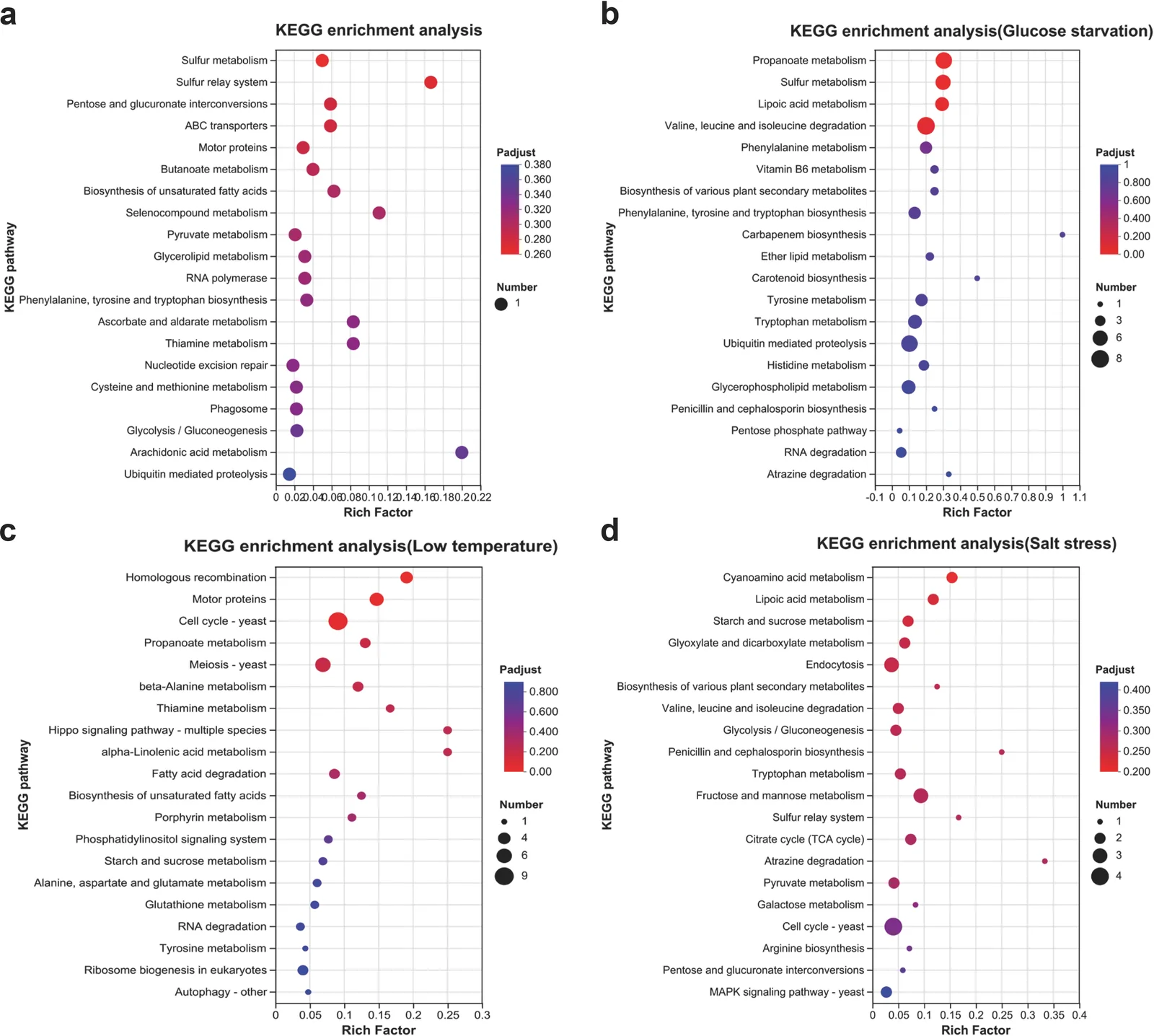
KEGG functional enrichment analysis of DEGs. KEGG enrichment analysis of (**a**) shared genes across three stresses, (**b**) glucose starvation-specific genes, (**c**) low-temperature-specific genes, and (**d**) salt stress-specific genes.

**Fig 8:**
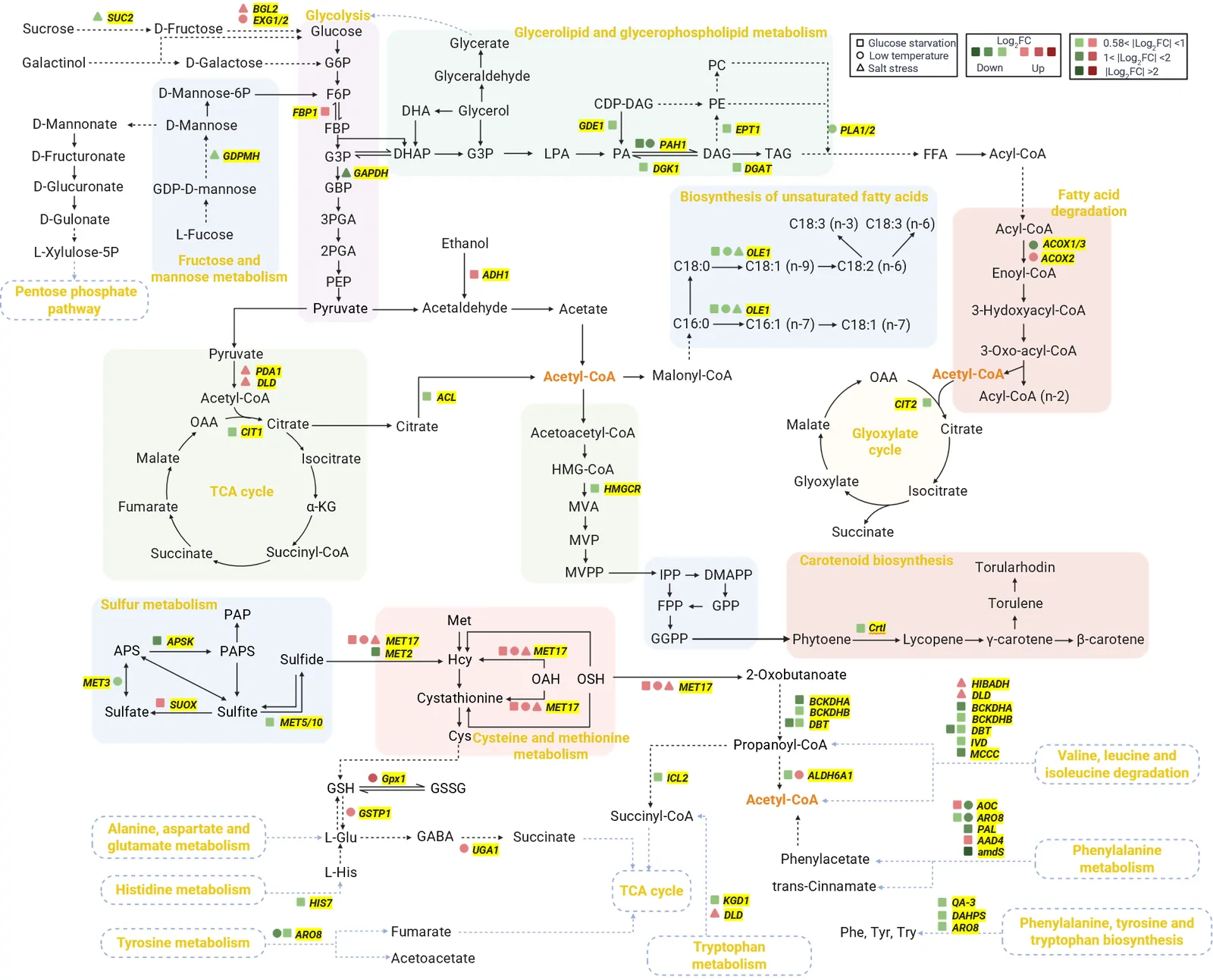
The potential metabolic pathways affected by RkMsn4 are illustrated. Information on the relevant genes is presented in Table S3. In the figure, squares represent glucose starvation, circles represent low temperature, and triangles represent salt stress. Dashed arrows indicate multiple steps, and solid arrows indicate single reaction steps. Genes with significant expression changes are highlighted in yellow.

To systematically characterize the regulatory roles of RkMsn4 under glucose starvation, low temperature, and salt stress, we first performed KEGG enrichment analysis on 64 shared DEGs and stress-specific DEGs across the 3 conditions (685 for glucose starvation, 326 for low temperature, and 184 for salt stress) (Fig. 7). Based on the KEGG annotation results, we further manually constructed an integrated metabolic network (Fig. 8), covering core pathways including carbohydrate metabolism, lipid metabolism, amino acid metabolism, carotenoid biosynthesis, terpenoid backbone biosynthesis, and glutathione metabolism.

In carbohydrate metabolism, *PDA1* (encoding the pyruvate dehydrogenase E1 component subunit α) and *DLD* (encoding dihydrolipoamide dehydrogenase), which are involved in pyruvate metabolism, showed significantly higher expression levels under salt stress. *CIT1*, which encodes citrate synthase in the TCA cycle, exhibited markedly lower expression levels under glucose starvation. Several key glycoside hydrolase genes associated with starch and sucrose metabolism as well as fructose and mannose metabolism also showed significantly lower expression levels under salt stress.

For lipid metabolism, *OLE1*, which encodes stearoyl-CoA 9-desaturase in the unsaturated fatty acid biosynthesis pathway, exhibited notably lower expression levels under all three stresses. Multiple genes related to glycerophospholipid metabolism were generally repressed during glucose starvation. *ACOX* encoding acyl-CoA oxidase in the fatty acid degradation pathway exhibited distinct expression changes under low temperature.

In amino acid metabolism, *MET17* (O-acetyl homoserine-O-acetyl serine sulfhydrylase), which participates in methionine and cysteine biosynthesis, showed significantly higher expression levels under all three stress treatments. A large number of genes involved in valine, leucine, and isoleucine degradation, as well as phenylalanine, tyrosine, and tryptophan metabolism, exhibited distinct expression changes under glucose starvation. *HIS7*, a key gene in the histidine biosynthesis pathway, also showed significantly lower expression levels under glucose starvation.

In addition, *CrtI*, which encodes phytoene desaturase in the carotenoid biosynthesis pathway, and *HMGCR*, which encodes 3-hydroxy-3-methylglutaryl-CoA reductase in the terpenoid backbone biosynthesis pathway, both showed significantly lower expression levels under glucose starvation. Two genes in glutathione metabolism, *GSTP1* (glutathione S-transferase) and *Gpx1* (glutathione peroxidase), showed significantly higher expression levels under low temperature.

### Construction of weighted co-expression networks and identification of key genes

**Fig 9:**
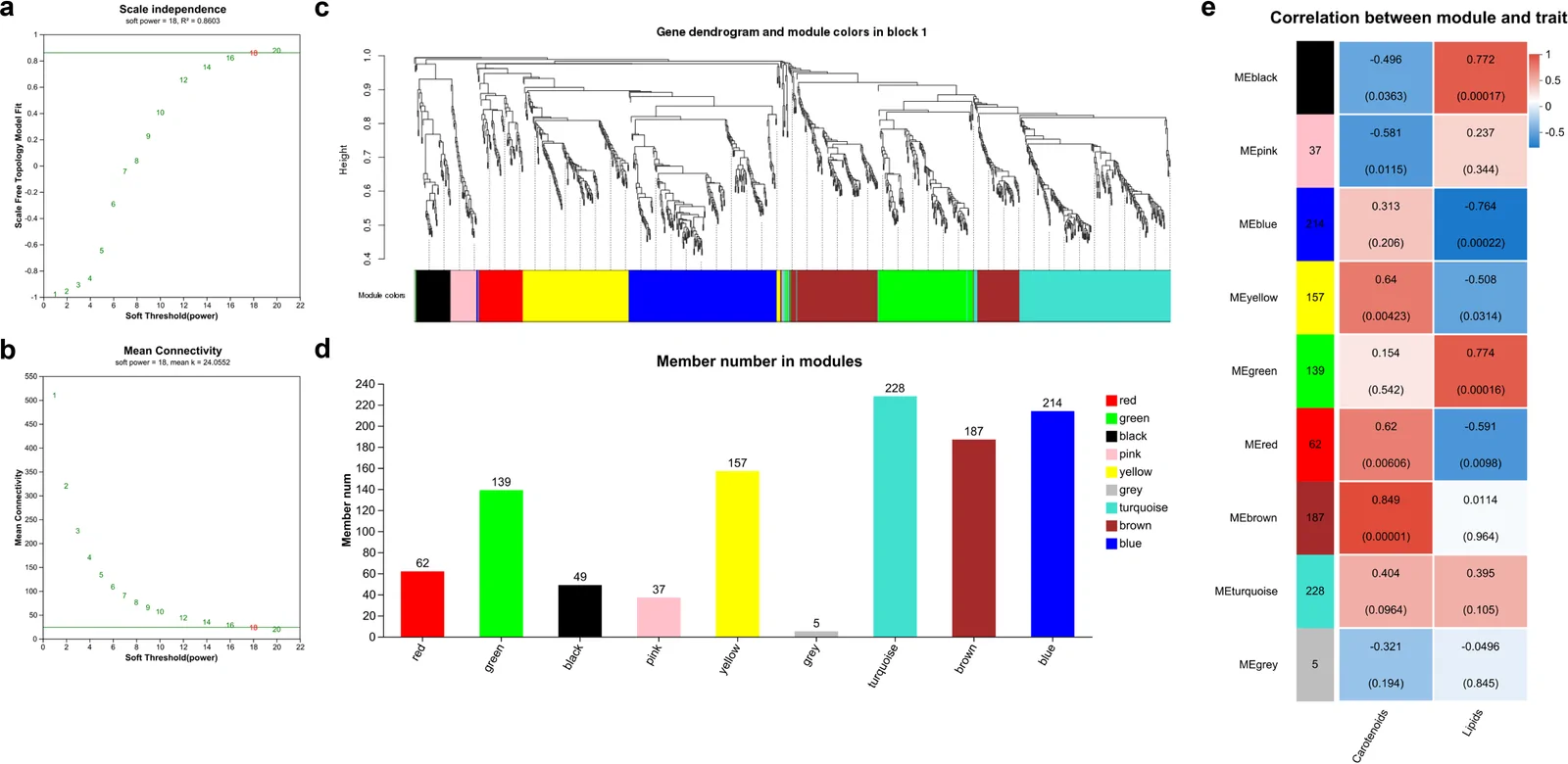
WGCNA analysis was performed. (**a**) The scale-free fit index curve is shown. The abscissa represents the soft threshold (β), whereas the ordinate represents the scale-free topology model fit index R2 for each soft threshold. (**b**) The scale-free mean connectivity curve is illustrated. The abscissa represents the soft threshold (β), whereas the ordinate represents the mean connectivity for each soft threshold. (**c**) Hierarchical clustering dendrogram of modules. (**d**) The number of DEGs in each module. (**e**) The correlation between modules and traits is shown. Module color representation of genes and their correlation with carotenoids and lipids.

To further explore the regulatory effects of RkMsn4 on carotenoid and lipid metabolism, the DEGs identified under the three stress conditions (1,484 genes in total) were assessed by WGCNA. Based on topology analysis, a soft-thresholding power of 18 was selected to construct a scale-free network (*R*^2^ = 0.8), with the average connectivity reduced to 24, which decreased redundant connections (Fig. 9a and b). As shown in Fig. 9c and d, genes were clustered into nine co-expression modules. The results of the module-trait correlation analysis indicated that the MEbrown module (*r* = 0.849, *P* = 0.00001) showed the strongest positive correlation with carotenoid metabolism, whereas the MEpink module (*r* = −0.581, *P* = 0.0115) showed the strongest negative correlation (Fig. 9e). For lipid metabolism, the MEgreen (*r* = 0.774, *P* = 0.00016) and MEblack (*r* = 0.772, *P* = 0.00017) modules showed the strongest positive correlations, while the MEblue module (*r* = −0.764, *P* = 0.00022) showed the strongest negative correlation (Fig. 9e). Although the MEturquoise module showed relatively lower correlations, it consistently showed positive associations with carotenoids and lipids in YM25235 (Fig. 9e).

To determine the functional relevance of genes within these key modules, KEGG enrichment analysis was performed (Fig. S3). The genes in the MEbrown module were enriched in pathways related to carotenoid biosynthesis, such as the synthesis of the terpenoid backbone, as well as other stress-response pathways. Genes in the MEpink module were enriched in competing pathways, including steroid biosynthesis and several pathways related to amino acid metabolism. The MEgreen and MEblack modules were enriched in pathways directly related to lipid metabolism, including glycerophospholipid metabolism, biosynthesis of unsaturated fatty acids, and fatty acid degradation. In contrast, the MEblue module was mainly enriched in pathways associated with carotenoid biosynthesis, cell growth, and proliferation, as well as cell wall biosynthesis.

**Fig 10:**
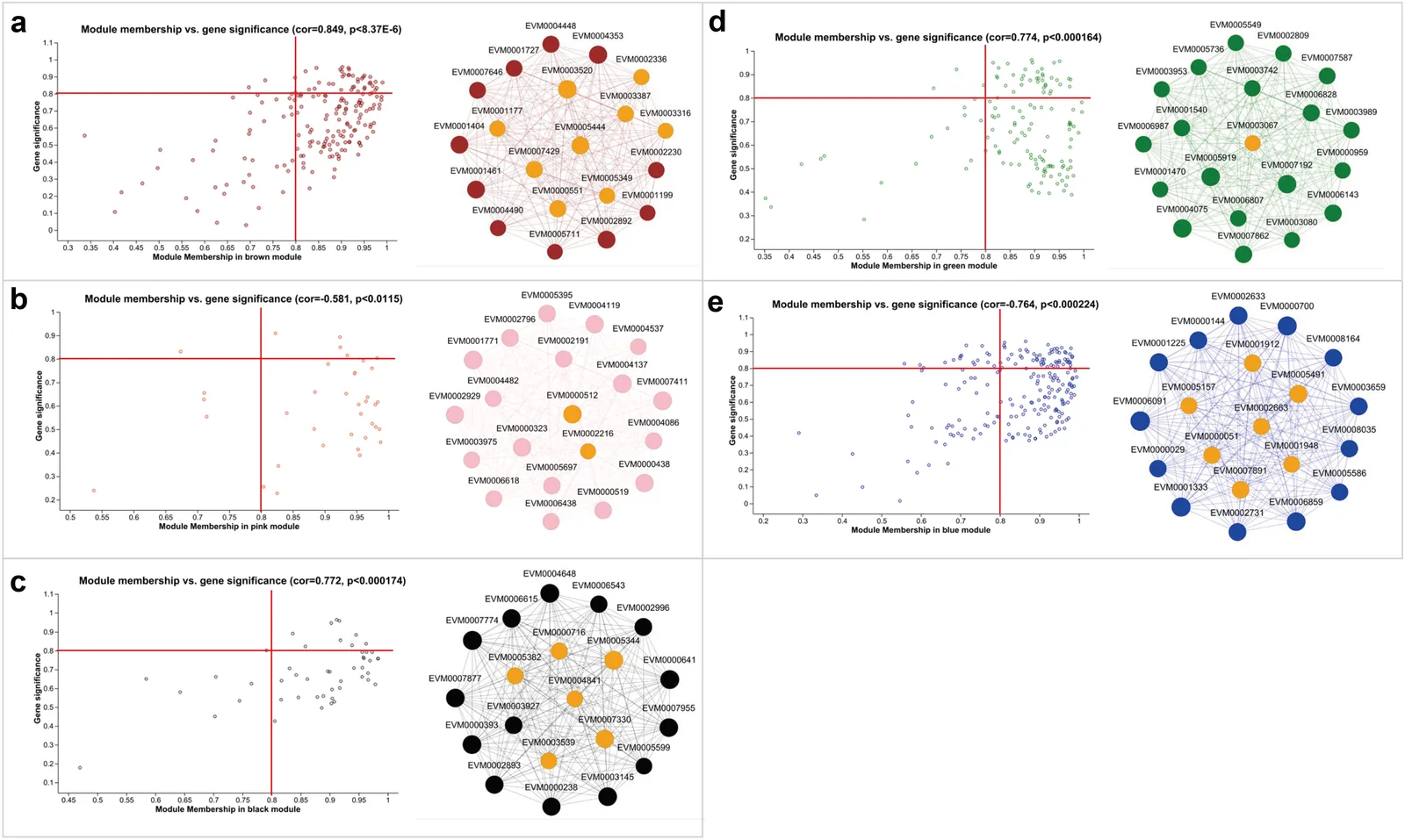
Identification of key genes based on the WGCNA. Key genes in (**a**) MEbrown, (**b**) MEpink, (**c**) MEblack, (**d**) MEgreen, and (**e**) MEblue modules were identified using MM-GS scatter plots and the top 20 hub genes ranked by intra-module connectivity.

We identified the key genes by first selecting candidate genes based on thresholds of |MM| > 0.8 and |GS| > 0.8 and overlapping them with the top 20 hub genes in each module (Fig. 10). Nine key genes were identified in the MEbrown module, including CAMK/CAMKL/PASK protein kinase, transcription factor MBZ1, and GDP-fucose protein O-fucosyltransferase (Fig. 10a and Table S4). Two key genes, both annotated as hypothetical proteins, were identified in the MEpink module (Fig. 10b and Table S4). Similarly, seven key genes were identified in both the MEgreen and MEblack modules, including phosphate phosphatase, 2-oxoacid dehydrogenase acyltransferase, and Pho85 cyclin-1 (Fig. 10c and d; Table S4). Seven key genes were identified in the MEblue module, including serine/threonine protein kinase Sid2, ATP adenylyltransferase, and PIF1 helicase (Fig. 10e and Table S4). Although the MEturquoise module consistently showed positive correlations with carotenoids and lipids, it’s overall associations with the phenotype were relatively weak (Fig. 9e). Therefore, the thresholds were relaxed (|MM| > 0.8 and |GS| > 0.4), which led to the identification of three more key genes, including dimethylaniline monooxygenase, and Mpv17/PMP22 family proteins (Table S4). Most of the identified genes contain STRE motifs, suggesting that they are potentially regulated by RkMsn4. These screened genes are potentially associated with carotenoid and lipid metabolism and can serve as candidates for subsequent functional validation.

## DISCUSSION

During fermentation, yeast cells are frequently exposed to various environmental stressors, which not only make the survival of cells challenging but also act as key triggers for the synthesis and accumulation of high-value secondary metabolites (4–6, 17). In industrial applications, improving yeast stress tolerance is a major bioengineering strategy to enhance the robustness and metabolic yield of industrial strains (30). In *S. cerevisiae*, the transcription factors Msn2/Msn4 serve as core regulators of gene expression in response to different types of environmental stressors (1, 8, 12). In this study, we found that deletion of *RkMsn4* in *R. kratochvilovae* YM25235 markedly weakened the strain’s tolerance and survival under salt, osmotic, oxidative, low temperature, heat, and glucose starvation (Fig. 1 and 2), demonstrating that RkMsn4 is an essential regulator for the environmental adaptability of YM25235. Notably, both the *RkMsn4* mutant and wild-type strain YM25235 exhibited severe growth inhibition at 35°C and 37°C (Fig. 1). This phenotype is likely associated with the low-temperature habitat where YM25235 evolved (45), which makes the strain thermosensitive and leads to obvious growth defects when the temperature rises above 32°C (Fig. S4). Collectively, RkMsn4 likely executes regulatory functions in YM25235 analogous to Msn2/Msn4 in *S. cerevisiae* and plays a critical role in responses to multiple environmental stresses. Given the native habitat characteristics of YM25235, investigating the regulatory mechanisms of RkMsn4 under glucose starvation, low temperature, and salt stress is of great significance.

Excessive intracellular ROS accumulation induced by diverse environmental stresses triggers oxidative damage and even cell death (49). Activation of the antioxidant system, involving enzymatic components (e.g., SOD and CAT) and non-enzymatic components (e.g., carotenoids and GSH), is a universal defensive strategy adopted by cells to rapidly counteract adverse stresses (8). Oxidative response patterns vary substantially across different stress types. Under glucose starvation, YM25235 clears excess ROS mainly through carotenoid accumulation and transcriptional activation of *RkCTT1* (47). When exposed to low temperature, the strain relies on synergistic antioxidant effects of carotenoids and GSH (46). By contrast, salt stress suppresses carotenoid biosynthesis, and cells enhance antioxidant capacity through higher expression levels of *RkCTT1* and *RkGpx1* (Fig. S5). Moderate salinity facilitates carotenoid production, while high salt concentration inhibits this pathway (39, 40); probably because cells preferentially redirect carbon flux toward synthesis of osmoprotectants (such as glycerol) and membrane lipid remodeling under hyperosmotic conditions (50, 51). Previous reports have confirmed that Msn2/Msn4 directly activates antioxidant enzyme genes including *CTT1* and *SOD1/2* to execute key antioxidant functions, and the double deletion of *Msn2* and *Msn4* renders cells sensitive to H_2_O_2_ (12, 19). Here, deletion of *RkMsn4* resulted in significantly lower expression levels of *RkCTT1*, accompanied by massive ROS accumulation under all three stress conditions (Fig. 3), further verifying the conserved and central function of Msn2/Msn4 in modulating *CTT1* expression. As essential non-enzymatic antioxidants in YM25235, carotenoid contents decreased in the *RkMsn4* deletion mutant under glucose starvation and low temperature, accompanied by reduced transcription of key synthetic genes *RkCrtI* and *HMGCR* (Fig. 4 and 8) although the transcriptomic data showed statistical significance only under glucose starvation. These results confirm that RkMsn4 positively participates in carotenoid biosynthesis regulation. To our best knowledge, this study is the first to establish a regulatory connection between Msn2/Msn4 and carotenoid synthesis, offering novel evidence for their antioxidant functions. Intriguingly, deletion of *RkMsn4* resulted in markedly higher expression levels of the cysteine synthetic gene *MET17* under all three stress treatments (Fig. 8). Cysteine is an indispensable precursor for GSH biosynthesis, and its bioavailability directly determines intracellular GSH levels (52). Transcriptomic data also revealed accelerated conversion of GSH to oxidized glutathione (GSSG) in the mutant under low temperature (Fig. 8). The increased reliance on the GSH antioxidant pathway suggests the activation of a compensatory antioxidant response, in which cells raise GSH demand to compensate for impairment of alternative antioxidant systems. Consistently, the higher expression level of *RkGpx1* in the *RkMsn4* mutant supports this hypothesis (Fig. 3). Gpx1 depends on GSH to detoxify intracellular H_2_O_2_, and its expression is not only regulated by Msn2/Msn4 but also induced by Snf1 kinase (53). Correspondingly, the *RkMsn4* mutant showed elevated GSH and GSSG pools under glucose starvation and accelerated conversion from GSH to GSSG under low temperature and salt stress (Fig. S6). In summary, although YM25235 adopts distinct antioxidant strategies under different stresses, RkMsn4 acts as a global stress regulator that modulates antioxidant responses to maintain cellular redox homeostasis in YM25235.

Membrane lipid remodeling is a crucial adaptive strategy that allows yeast cells to cope with environmental stress and maintain cellular homeostasis (29, 34, 36, 54, 55). Remodeling lipid compositions (e.g., UFAs and ergosterol) enables improved tolerance against ethanol, salt, and other stresses (35). Upon glucose starvation, YM25235 catabolizes lipids via β-oxidation to generate acetyl-CoA and energy (47). Under low temperature, it increases the proportion of UFAs to maintain the fluidity of membranes (48). Under salt stress, accumulation of large amounts of lipids was observed in YM25235 (Fig. S7), possibly due to redirected carbon flux toward osmolyte synthesis (e.g., glycerol), which indirectly increases the precursors of lipids (50, 51). In *S. cerevisiae*, Msn2/Msn4 positively controls genes involved in fatty acid β-oxidation to combat carbon limitation, and double deletion of *Msn2* and *Msn4* leads to the accumulation of non-polar lipids (triacylglycerols [TAG] and sterol esters [SE]) (24, 25). Differently, our results demonstrated that deletion of *RkMsn4* resulted in significantly lower expression levels of multiple core genes governing fatty acid degradation and synthesis, including *RkACOX2*, *RkACAA1*, *RkFAS1,* and *RkACCS* (Fig. 5). Consequently, total lipid content increased in the mutant under glucose starvation but declined under low temperature (non-significant) and salt stress (Fig. 5), implying a possible complicated and condition-dependent regulation of lipid metabolism by RkMsn4. Notably, RkMsn4 exerts prominent control over lipid composition especially under glucose starvation (Fig. 8). Specifically, deletion of *RkMsn4* resulted in significantly lower expression levels of genes mediating the conversion of phosphatidic acid (PA) to phospholipids (e.g., phosphatidylcholine [PC] and phosphatidylethanolamine [PE]) and glycerolipids (DAG and TAG), including phosphate phosphatase (*PAH1*), ethanolamine phosphotransferase (*EPT1*), and diacylglycerol acyltransferase (*DGAT*). Meanwhile, genes encoding diacylglycerol kinase (*DGK1*, responsible for DAG to PA conversion) and glycerophosphodiesterase (*GDE1*) also showed markedly lower expression levels. PA acts not only as a pivotal precursor for phospholipid and glycerolipid production but also as a signaling lipid modulating signal transduction and organelle interaction (56, 57). Therefore, RkMsn4 may drive membrane lipid remodeling under stress by regulating the substrate cycle between PA and DAG. Additionally, yeast generally accumulates UFAs to sustain membrane fluidity and antioxidant capacity under different types of stress (29, 34, 36, 54, 55). In this study, UFAs content especially monounsaturated fatty acids dropped in the *RkMsn4* deletion mutant under all three stresses (Table 1). Transcriptomic analysis revealed significantly lower expression levels of the Δ9-desaturase gene *OLE1* under all three stresses (Fig. 8), whose encoded enzyme catalyzes the initial step converting SFAs to UFAs (58). These results highlight that RkMsn4 plays a key role in regulating fatty acid unsaturation and membrane adaption. Thus, RkMsn4 modulates lipid homeostasis via sophisticated compositional adjustment rather than simply promoting or suppressing lipid degradation/synthesis. Future integrative lipidomic profiling will deepen our understanding of Msn2/Msn4-mediated lipid regulatory network.

Particularly, RkMsn4 exhibits prominent regulatory effects on amino acid metabolism specifically under glucose starvation. The deletion of *RkMsn4* resulted in significantly lower expression levels of multiple genes in the branched-chain amino acid (BCAA; leucine, isoleucine, and valine) degradation pathway (Fig. 8), including genes encoding branched-chain α-keto acid dehydrogenase complex (*BCKDHA* and *BCKDHB*), 2-oxoacid dehydrogenase acyltransferase (*DBT*), acyl-CoA dehydrogenase (*IVD*), methylcrotonyl-CoA carboxylase (*MCCC*), and methylmalonate semialdehyde dehydrogenase (*ALDH6A1*), accompanied by significant accumulation of valine and isoleucine (Fig. S8). BCAAs play essential roles in abiotic stress adaptation across plants and microorganisms (59), and our previous work also verified their critical functions in YM25235 responding to glucose starvation (47). The degradation of BCAAs generates acetyl-CoA and succinyl-CoA, which feed into the TCA cycle or gluconeogenesis, thereby supporting metabolic homeostasis under nutrient-limited conditions (47). Moreover, RkMsn4 significantly shapes aromatic amino acid (AAA; phenylalanine, tryptophan, and tyrosine) metabolism (Fig. 8). Key biosynthetic genes (DAHP synthase *DAHPS* and quinate dehydrogenase *QA-3*) and catabolic genes (L-phenylalanine ammonia-lyase *PAL* and aromatic amino acid transaminase *ARO*) were significantly repressed, resulting in intracellular enrichment of tryptophan and phenylalanine (Fig. S8). Tryptophan contributes to the stability of membranes and enhances tolerance to temperature, ethanol, oxidative stress, and organic acids (60, 61), while phenylalanine supports carbon-nitrogen balance through degradation and transamination (62). Accordingly, RkMsn4 probably facilitates the adaptation of the YM25235 strain to glucose starvation by modulating central carbon and nitrogen fluxes through the regulation of BCAA and AAA metabolism.

Osmoregulation constitutes a fundamental adaptive mechanism for cells coping with environmental stresses, especially water imbalance triggered by salt stress (49). In *S. cerevisiae*, Msn2/Msn4 enhances salt tolerance by regulating the biosynthesis of osmoprotectants such as glycerol, trehalose, and proline, and the trehalose synthesis-related genes (*TPS1* and *TPS2*) have been validated as direct targets of Msn2/Msn4 (16, 20–22). Nevertheless, deletion of *RkMsn4* barely altered the expression of these canonical osmotic-regulation-related genes in this study, and the intracellular trehalose level decreased slightly without statistical significance (Fig. S9). These results indicate that RkMsn4 has a relatively limited direct impact on endogenous osmoprotectant synthesis in YM25235, whereas such function is probably dominated by Hot1, a transcription factor specifically responsible for glycerol production (49). Unlike intracellular osmolyte biosynthesis, deletion of *RkMsn4* resulted in markedly lower expression levels of genes responsible for exogenous sugar utilization, including β-fructofuranosidase gene *SUC2* and multiple glycoside hydrolase genes (Fig. 8). Repression of these genes probably impedes hydrolysis, transport, and uptake of extracellular sucrose and fructose. In plants and microorganisms, soluble sugars, such as glucose, trehalose, sucrose, and fructose, serve as carbon sources and function as osmotic regulators and antioxidants, accumulating to alleviate osmotic stress and increase salt tolerance (63, 64). Therefore, RkMsn4 indirectly maintains osmotic homeostasis under salt stress by modulating acquisition and utilization of exogenous sugars.

Collectively, our results demonstrated that RkMsn4 significantly affects multiple metabolic processes during stress adaptation in YM25235, among which carotenoid and lipid metabolism attract intensive research interest. These two classes of metabolites not only constitute core components of the stress response machinery in the YM25235 but also represent high-value products in red yeasts. We further investigated the regulatory roles of RkMsn4 in carotenoid and lipid metabolism via WGCNA and identified a set of key candidate genes potentially regulated by RkMsn4 and correlated with the metabolism of carotenoids and lipids (Fig. 9 and 10 and Table S4). For instance, the protein kinase PASK, which senses the nutrient status and regulates the distribution of glucose carbon flow (65); the transcription factor MBZ1, which regulates the integrity of the cell wall (66); and protein O-fucosyltransferase (POFUT), which is involved in protein glycosylation modification (67). Although these genes are not directly linked to carotenoid biosynthesis, they are presumed to perform vital physiological functions in YM25235. Additionally, *PAH1*, a key gene associated with lipid metabolism, was identified, which aligns with our earlier discussion. The *DBT* gene involved in the degradation of BCAAs may also contribute to lipid metabolism, given that BCAAs and lipid synthesis are closely related (68). Several genes involved in carotenoid and lipid metabolism were also identified, such as dimethylaniline monooxygenase, which is related to redox reactions (69), and the Mpv17/PMP22 family protein (homologous to Sym1 in *S. cerevisiae*), which is involved in heat and ethanol stress and the transport of TCA cycle intermediates (70). Our preliminary verification indicated that *Sym1* deletion reduces both carotenoid and lipid accumulation (data to be published elsewhere). Though most abovementioned genes have not been experimentally validated to directly participate in carotenoid or lipid metabolism, their tight phenotypic correlation highlights their prospective pivotal roles in stress adaptation and metabolic regulation of YM25235.

Several limitations remain in this study. Restricted by the research schedule, functional verification of the regulatory relationship between RkMsn4 and key candidate genes is still in progress. Meanwhile, subsequent studies focus on the protein expression and phosphorylation modification of RkMsn4. In addition, only three stress conditions (glucose starvation, low temperature, and salt stress) were analyzed in this work, so additional physiological roles of RkMsn4 under other stressors await further exploration.

## MATERIALS AND METHODS

### Yeast strains and culture conditions

Three yeast strains were employed in this study: wild-type *R. kratochvilovae* YM25235, the *RkMsn4* deletion mutant YM25235RkMsn4Δ, and the complemented strain YM25235RkMsn4Δ/pRZRkMsn4. The mutant and complemented strains were constructed following procedures described in a previous report (71). Briefly, the deletion strain was generated via the CRISPR-Cas9 gene-editing system. First, 20-nucleotide gRNAs were designed using an online CRISPR platform (http://crispor.tefor.net/) based on the genomic sequence of *RkMsn4* from the *R. kratochvilovae* YM25235 genome (NCBI accession number: PRJNA739038), to prepare sgRNA expression constructs (Table S5). Meanwhile, the full-length coding sequence of *RkMsn4* was amplified from YM25235 cDNA and subcloned into the pRZ2034 vector to generate the complementation plasmid pRZRkMsn4. Subsequently, the sgRNA transcription plasmid was introduced into *R. kratochvilovae* YM25235 (Ura3∆) by *Agrobacterium tumefaciens*-mediated transformation. The *RkMsn4* deletion mutants were screened on SCD-Ura medium and verified by PCR and sequencing (Table S6 and Fig. S10). Furthermore, the complementation plasmid was transformed into the *RkMsn4* deletion mutant using the same transformation method. Positive complemented strains were selected on YPD plates supplemented with 11.25 µg/mL Zeocin and finally confirmed by sequencing.

Four culture media were prepared as follows: yeast extract-peptone-dextrose (YPD; 1% yeast extract, 2% peptone, and 2% glucose), glucose-free YP medium (1% yeast extract and 2% peptone), synthetic complete medium (SCD; 0.17% yeast nitrogen base, 2% glucose, 0.5% ammonium sulfate, and amino acid mixture, uracil added or removed as required), and glucose-free SC medium (0.17% yeast nitrogen base, 0.5% ammonium sulfate, and amino acid mixture).

### Spot assay, survival rate, and growth curve measurement

To conduct a spot assay, yeast cells were grown overnight at 28°C in YPD liquid medium to the early logarithmic phase, collected and washed twice with ddH_2_O, and adjusted to OD_600_ = 1.0. Next, 10-fold serial dilutions were spotted on YPD plates supplemented with NaCl (0.75–1.25 M), sorbitol (1.2–1.7 M), or H_2_O_2_ (4–6 mM). Glucose starvation was assessed using glucose-free YP and SC media. To induce temperature stress, diluted cells were spotted on YPD plates and incubated at 15°C, 32°C, 35°C, and 37°C, with 28°C used as the control condition. All treatments were performed in parallel, and colony growth was monitored after 2–5 days.

To determine the survival rate, yeast cells were exposed to different types of stress. The cells were treated with salt (NaCl, 1–5 M), osmotic (sorbitol, 1–3 M), oxidative (H_2_O_2_, 10–80 mM), and heat shock (35–50°C) for 30 min. The cells were also exposed to low temperature (15°C, 0–48 h) and glucose starvation (glucose-free YP and SC media, 0–72 h) treatments. After treatment, diluted cells were plated on YPD plates and incubated at 28°C for 24 h, followed by colony-forming unit (CFU) counting. Survival rate was calculated as CFU of treated cells relative to the control (72). For low temperature and glucose starvation treatments, the results were expressed as the CFU ratio of the *RkMsn4* deletion mutant to the YM25235 strain to minimize the effect of cell proliferation.

Growth curves were determined under three stress conditions. For glucose starvation, cells were cultured in 100 mL YPD medium at 28°C with shaking at 160 rpm for 168 h to monitor cell growth upon glucose depletion. For low temperature, strains were incubated in 100 mL YPD at 15°C and 160 rpm over 168 h. For salt stress, a range of NaCl concentrations was first screened in YPD (Fig. S11), and 4% (wt/vol) NaCl with negligible adverse effects on cell growth was selected as the working stress concentration for subsequent growth assessment. Samples were collected every 24 h for dry weight measurement (DCW).

### Determination of intracellular ROS levels

Intracellular ROS content was determined as previously described (47). Briefly, a defined amount of cells (5 × 10^6^) was harvested, stained with 2′,7′-dichlorodihydrofluorescein diacetate (DCFH-DA) (Beyotime, Shanghai, China), and measured on an Infinite M200 PRO microplate reader (Tecan, Männedorf, Switzerland) at excitation/emission wavelengths of 488/525 nm.

### Determination of total carotenoids, lipids, and fatty acids

As described in this study, yeast cells were harvested after cultivation under the three stress conditions. Cells were collected by centrifugation at 4,500 rpm for 5 min at 4°C, washed with PBS, and lyophilized. The contents of carotenoids, lipids, and fatty acids were determined using methods reported in previous studies (43, 71). For total carotenoid extraction, 0.4 g of dry cells was ground with SiO_2_, and carotenoids were repeatedly extracted with acetone-methanol at room temperature. The supernatant was collected, and the total carotenoid content was determined by measuring the optical density at 450 nm, where β-carotene is the major contributor (73).

Total lipids were extracted using the Bligh-Dyer method (74). Briefly, 4 mol/L HCl was added to 0.4 g dry cells and mixed thoroughly and incubated at room temperature for 30 min. The mixture was boiled in a water bath for 3 min and cooled to room temperature. Chloroform/methanol (2:1, vol/vol) was subsequently added, followed by centrifugation at 8,000 rpm for 5 min. The organic phase was transferred to a clean tube, and lipid extraction was repeated twice. The combined extracts were dried and weighed to calculate lipid content.

Fatty acids were methyl-esterified using BF_3_-MeOH. Briefly, 10% KOH was added to 0.1 g dry cells for fatty acid extraction, followed by saponification at 70 °C for 3–5 h. The sample pH was adjusted to 2.0 with HCl. The extracted fatty acids were methyl-esterified in 14% BF_3_-MeOH solution at 70°C for 1.5 h. Fatty acid methyl esters were then extracted with hexane and analyzed by gas chromatography-mass spectrometry (GC-MS) (75).

### Sequence analysis

Gene sequences were retrieved from the *R. kratochvilovae* YM25235 genome database (NCBI accession number: PRJNA739038), and a 2,000 bp fragment upstream of the start codon (ATG) of each target gene was defined as its promoter region. The occurrence of STRE motifs (A_4_G: 5′-AGGGG-3′ and C_4_T: 5′-CCCCT-3′) within these promoter sequences was subsequently counted.

### RNA isolation, sequencing, and bioinformatics analysis

Samples under glucose starvation and low temperature were harvested following our previously published protocols (46, 47). For salt stress samples, yeast cells were cultured in liquid YPD supplemented with 4% NaCl until stationary phase. All cells were pelleted by centrifugation at 4°C and 4,500 rpm for 5 min, followed by two washes with PBS. Total RNA was extracted, libraries were constructed, and sequencing was performed by Majorbio (Shanghai, China), and the raw data obtained were deposited in the NCBI database (accession number: PRJNA1405149). Clean reads were mapped to the reference genome (NCBI accession number: PRJNA739038) using HISAT2, and gene expression was quantified as TPM. Differentially expressed genes (DEGs) were identified using *p*-adjust < 0.05 and |fold change| ≥ 1.5. Using these DEGs, we performed pathway enrichment analysis using the Kyoto Encyclopedia of Genes and Genomes (KEGG).

### Weighted gene co-expression network analysis and identification of key genes

We performed WGCNA on the identified DEGs to construct co-expression networks. Co-expression modules were generated using an automatic network construction algorithm, and modules significantly associated with the metabolism of carotenoids and lipids were selected for KEGG pathway enrichment analysis. We screened candidate genes based on gene significance (GS) and module membership (MM). The intersection of these candidates with the top 20 most highly connected hub genes in each module was defined as the set of key genes.

### Quantitative real-time PCR analysis

qRT-PCR analysis was further applied to verify the transcriptional changes of genes involved in carotenoid biosynthesis, lipid metabolism, and antioxidant caused by *RkMsn4* deletion. The primer sequences used in this study are listed in Table S7. Small subunit rRNAs (*SSU rRNAs*) were used as the endogenous reference gene. We performed qRT-PCR analysis using ChamQ SYBR qPCR Master Mix (Vazyme, Nanjing, China) on the Bio-Rad CFX96 Fast Real-Time PCR System (Bio-Rad, Hercules, CA, USA) following the manufacturer’s instructions. Relative levels of transcripts were quantified using the 2^−ΔΔCT^ method. To validate the reliability of RNA-seq data, the log_2_(fold change) values of genes related to carotenoid biosynthesis, lipid metabolism, and antioxidant defense were compared between qRT-PCR and RNA-seq results.

### Determination of glutathione, amino acids, and trehalose

Reduced glutathione (GSH) and oxidized glutathione (GSSG) were extracted and quantified with commercial kits (BOXBIO, Beijing, China) following the manufacturer’s protocols, and their contents as well as the GSH/GSSG ratio were calculated.

Intracellular amino acid contents of yeast were quantified by an optimized high-performance liquid chromatography (HPLC) method (76). Briefly, amino acids were ultrasonically extracted from yeast cells using 0.1 mol/L HCl in a water bath, followed by derivatization with 1% 2,4-dinitrofluorobenzene (DNFB). After filtration through a 0.22 μm membrane, samples were kept at 4°C before measurement. Chromatographic analysis was carried out on an Agilent 1260 Infinity II and an Agilent Zorbax SB-C18 (250 mm × 4.6 mm, 5 μm) column. Mobile phase A was 0.05 mol/L NaAc (pH 6.8) containing 1% *N*,*N*-dimethylformamide, and mobile phase B was acetonitrile–water (1:1, vol/vol). A linear gradient was programmed to increase the acetonitrile content to 50%. The detection wavelength was set at 360 nm, column temperature at 25°C, flow rate at 1 mL/min, and injection volume at 20 μL.

Trehalose content in yeast was determined via the anthrone-sulfuric acid colorimetric method as previously described (77). Briefly, samples were repeatedly extracted with 0.5 mol/L trichloroacetic acid in an ice-water bath for 45 min per extraction. Subsequently, 0.2% anthrone-sulfuric acid reagent was added to the extracts, followed by incubation in a boiling water bath for 10 min. After cooling, absorbance was measured at 620 nm. A trehalose standard curve was plotted to calculate the regression equation and regression coefficient (*R*^2^).

### Statistical analysis

Statistical analysis and data visualization were performed using GraphPad Prism 9.5 (GraphPad Software, CA, USA). The data were presented as the mean ± standard deviations (SDs) from at least three independent experiments. The differences between groups were determined by conducting the Tukey test (*P < 0.05, **P < 0.01, and ***P < 0.001).

## Data Availability

The raw sequencing data have been deposited in the NCBI database and are publicly available under accession number PRJNA1405149.

## SUPPLEMENTAL MATERIAL

Supplemental figures aem.00647-26-s0001.pdf Fig. S1 to S11.

Supplemental tables aem.00647-26-s0002.pdf Tables S1, S2, and S4 to S7.

Table S3 aem.00647-26-s0003.xlsx DEGs under glucose starvation, low temperature, and salt stress.

ASM does not own the copyrights to Supplemental Material that may be linked to, or accessed through, an article. The authors have granted ASM a non-exclusive, world-wide license to publish the Supplemental Material files. Please contact the corresponding author directly for reuse.
