# Supplementary material for: RkMsn4, a zinc finger transcription factor, regulates carotenoid and lipid metabolism to enhance tolerance to environmental stress in Rhodosporidium kratochvilovae
Source: Appl Environ Microbiol. 2026 Sep 23;92(10):e00647-26. doi: 10.1128/aem.00647-26 (PMC13689428; doi:10.1128/aem.00647-26)
Supplement: Supplementary file 1 [file aem.00647-26-s0001.pdf]

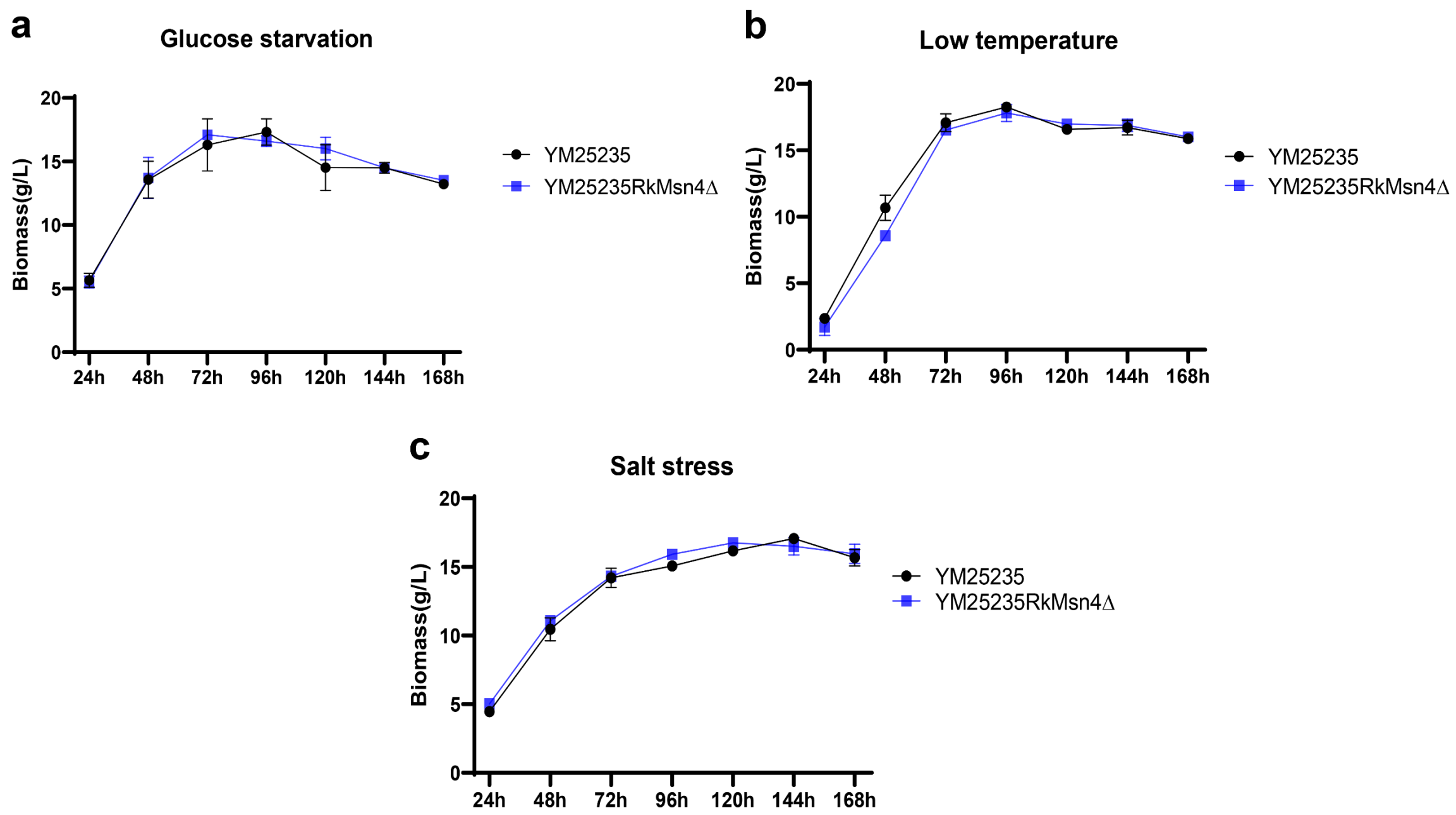

Fig. S1. Growth curves of the *RkMsn4* deletion mutant under **(a)** glucose starvation, **(b)** low temperature and **(c)** salt stress.

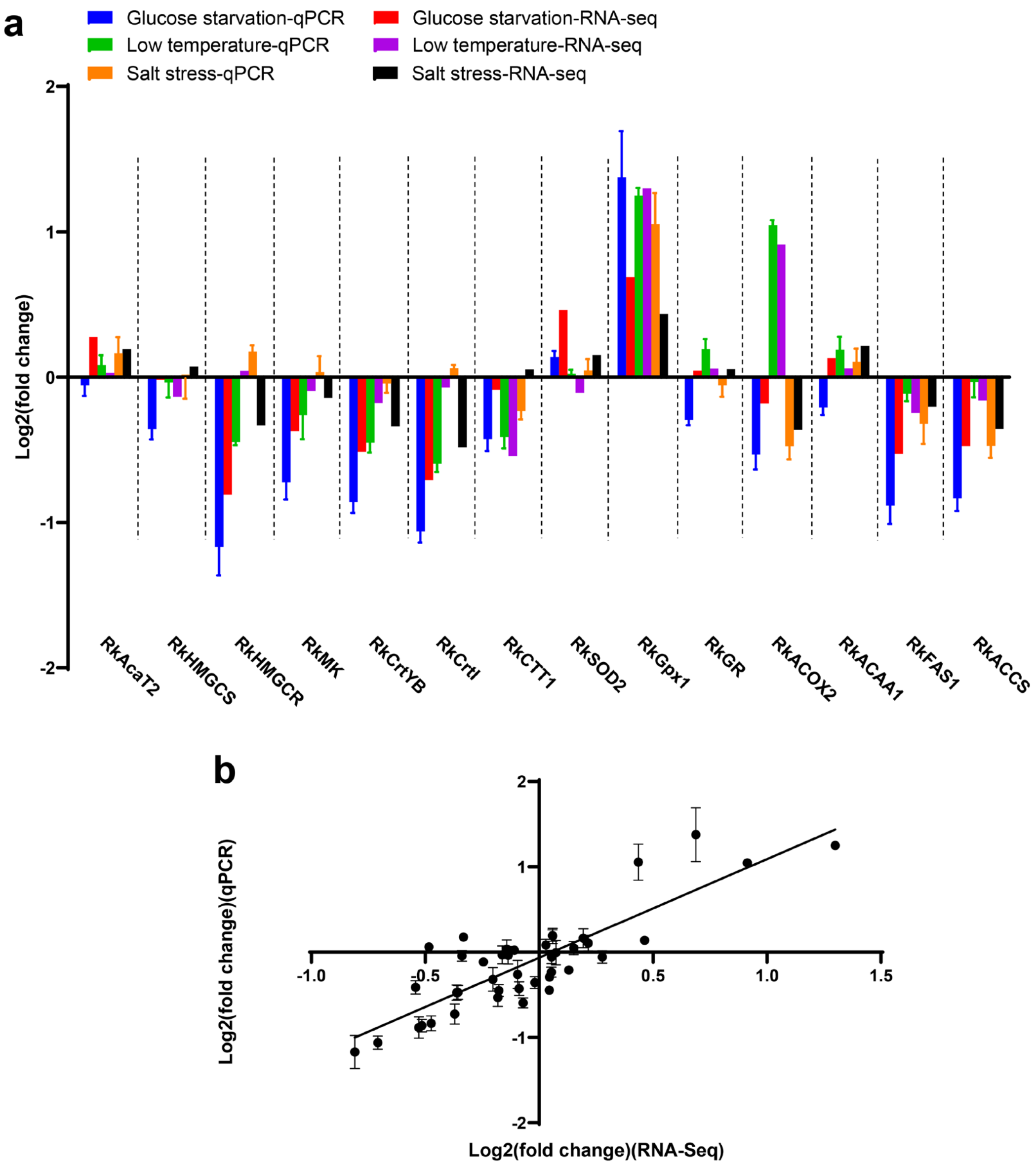

Fig. S2. Confirmation of RNA-seq results by qRT-PCR assay. **(a)** Comparison of the fold changes in expression levels of selected genes detected by RNA-seq and qRT-PCR. **(b)** Correlation of gene expression fold changes between RNA-seq and qRT-PCR analyses. Pearson's correlation coefficient = 0.7011, p <0.0001.

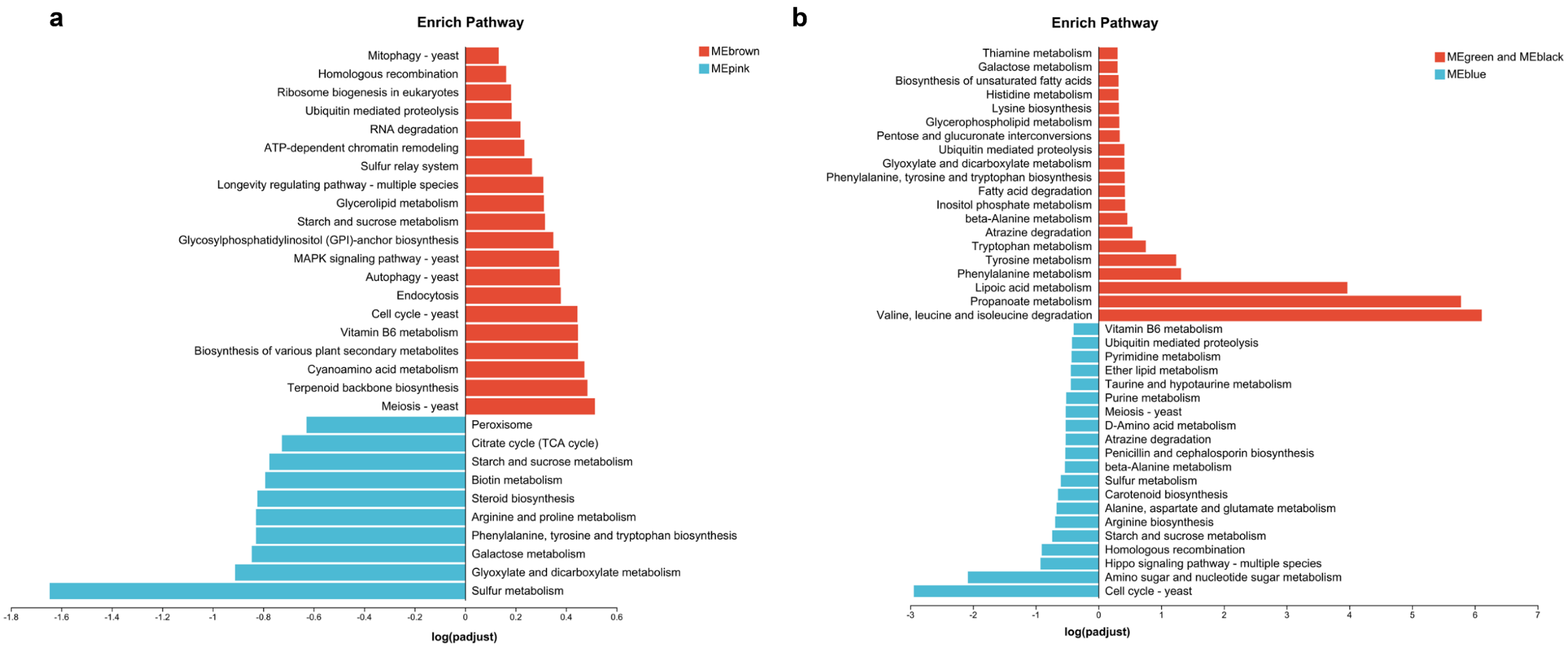

Fig. S3. KEGG pathway enrichment analysis of module genes. **(a)** KEGG enrichment analysis of genes in the MEbrown and MEpink modules. **(b)** KEGG enrichment analysis of genes in the MEgreen, MEblack, and MEblue modules. The vertical axis represents pathway names. For the horizontal axis, values greater than zero are defined as -$\log_{10}$(padjust), while values less than zero are defined as $\log_{10}$(padjust), enabling simultaneous visualization of the bar charts for two gene sets.

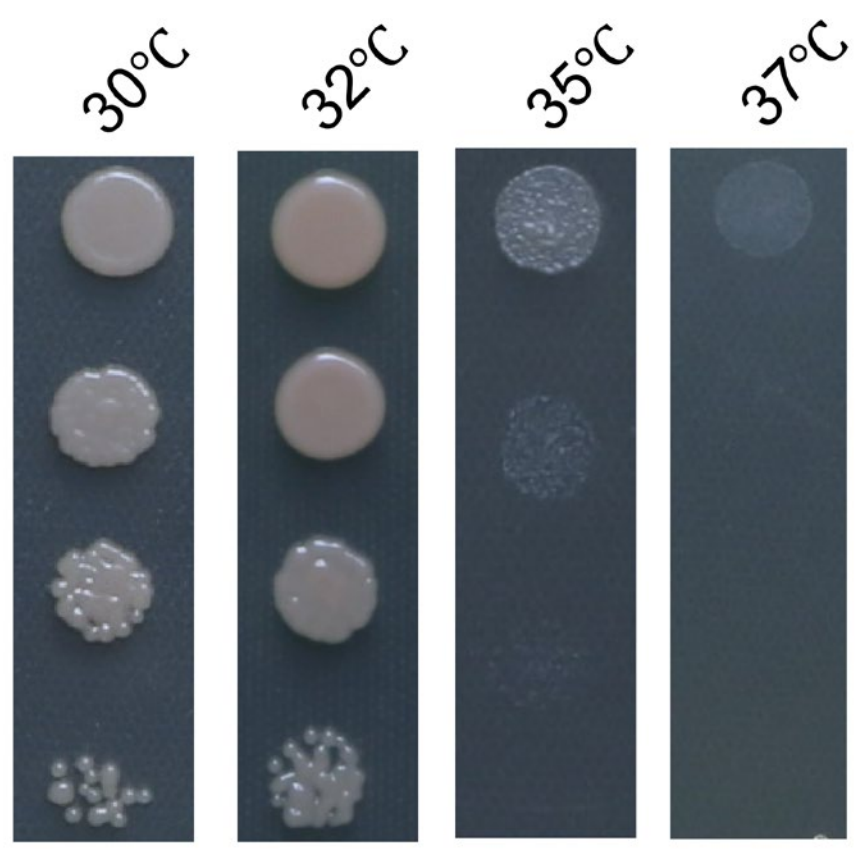

Fig. S4. Growth status of YM25235 under heat stress.

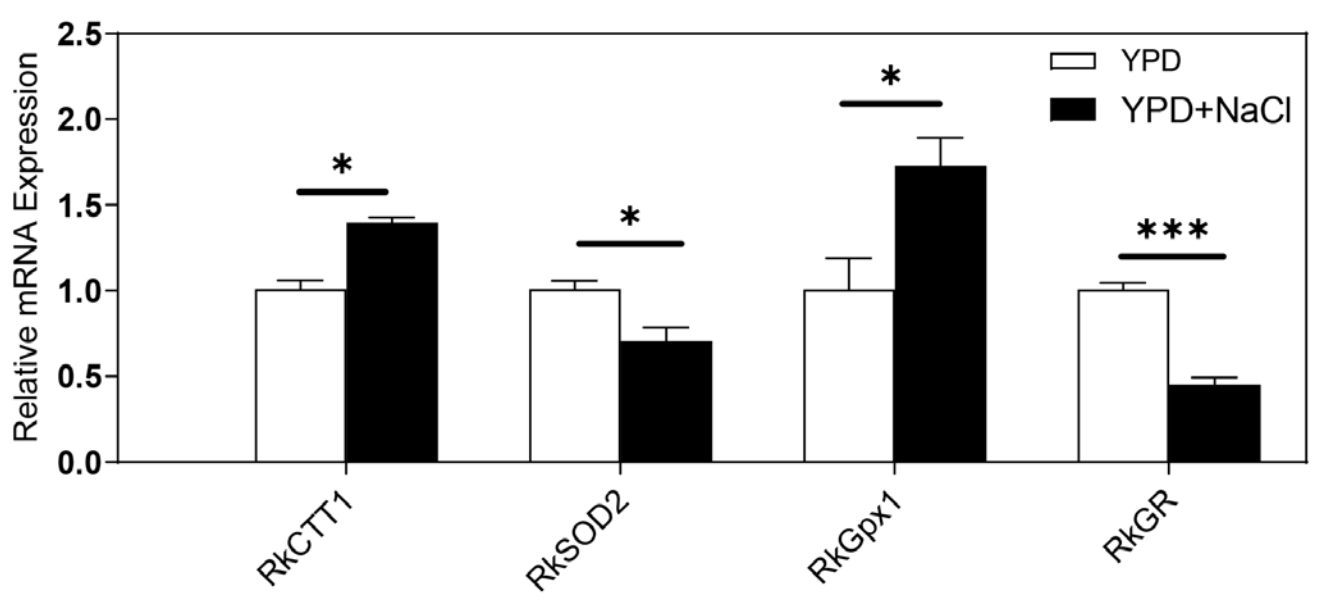

Fig. S5. Expression of antioxidant enzyme genes in YM25235 under salt stress. *RkCTT1*: catalase

gene; *RkSOD2*: superoxide dismutase gene; *RkGpx1*: glutathione peroxidase 1 gene; *RkGR*: glutathione reductase gene.

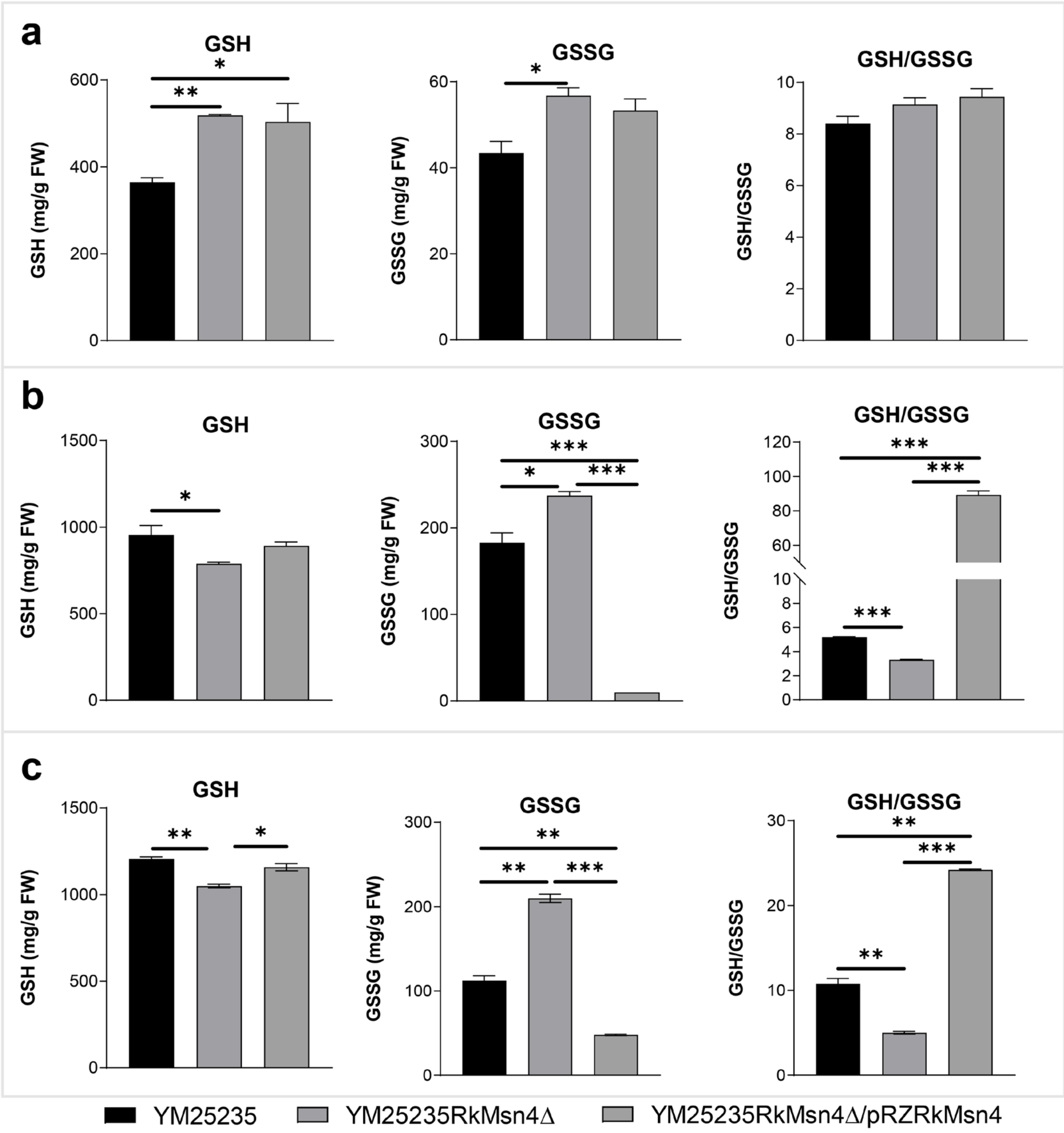

Fig. S6. Analysis of glutathione contents in the strains under **(a)** glucose starvation, **(b)** low temperature and **(c)** salt stress. GSH: reduced glutathione; GSSG: oxidized glutathione.

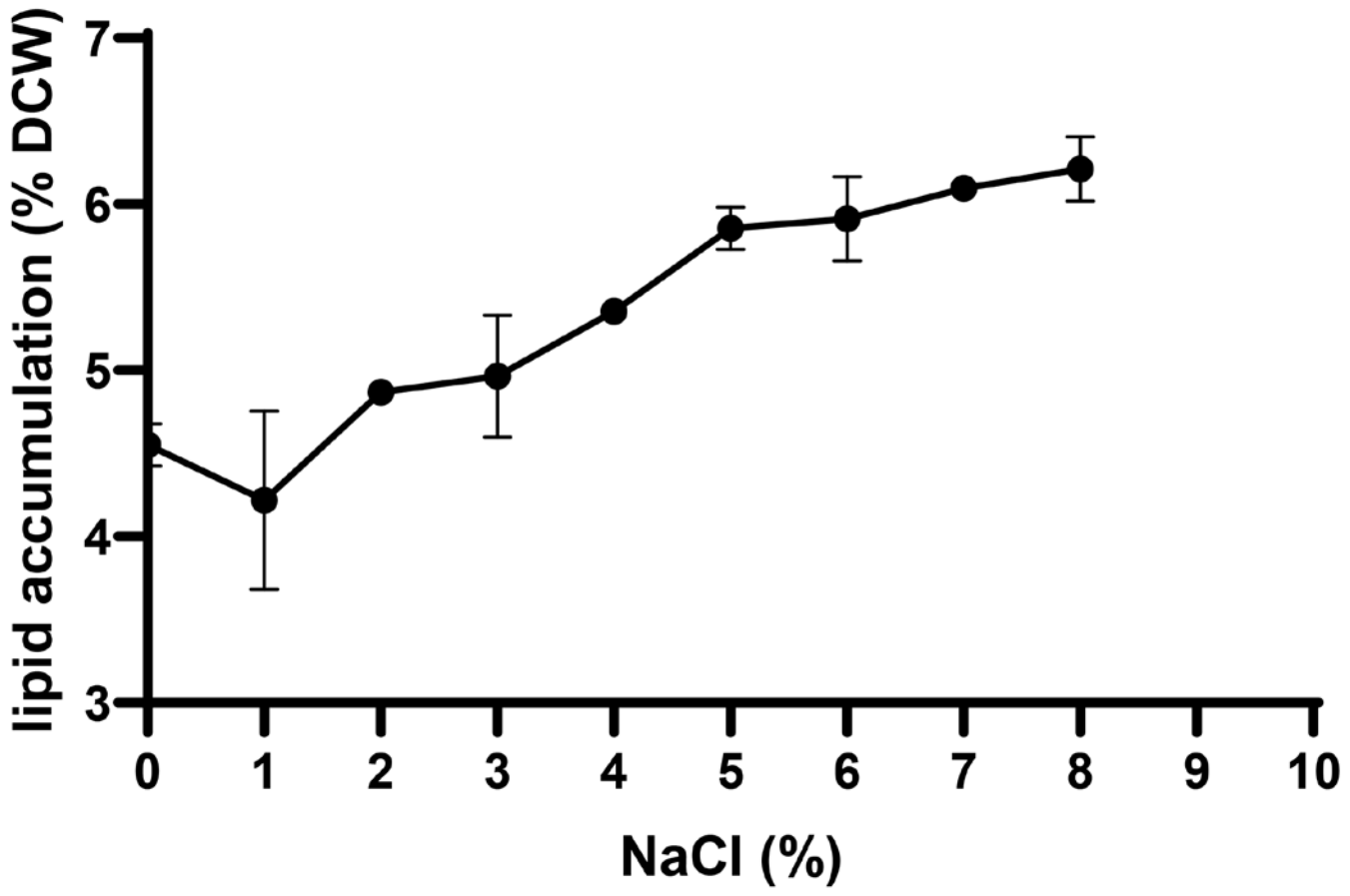

Fig. S7. Lipid content in YM25235 under salt stress.

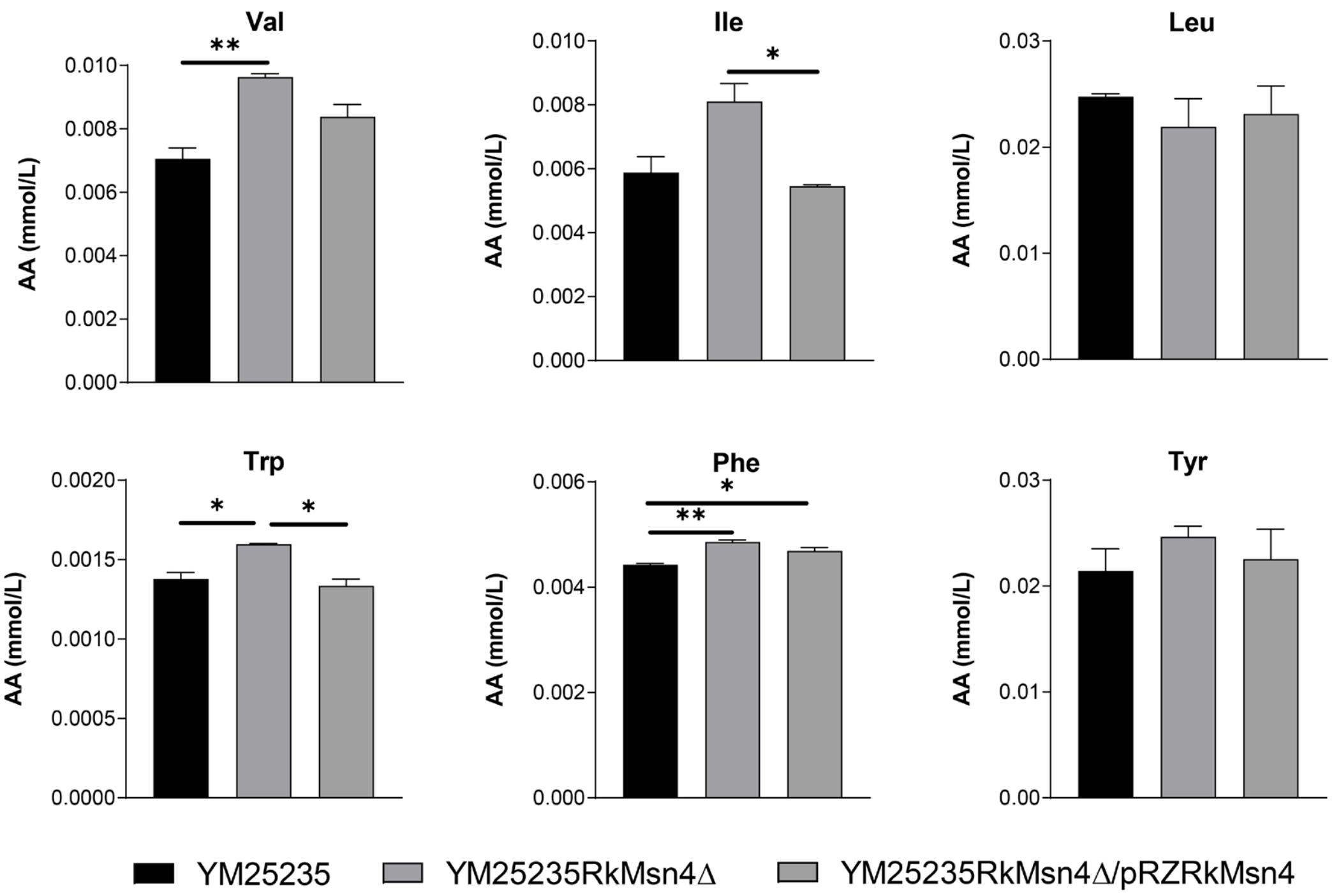

Fig. S8. Analysis of amino acid contents under glucose starvation. Val: Valine; Ile: Isoleucine; Leu: Leucine; Trp: Tryptophan; Phe: Phenylalanine; Tyr: Tyrosine.

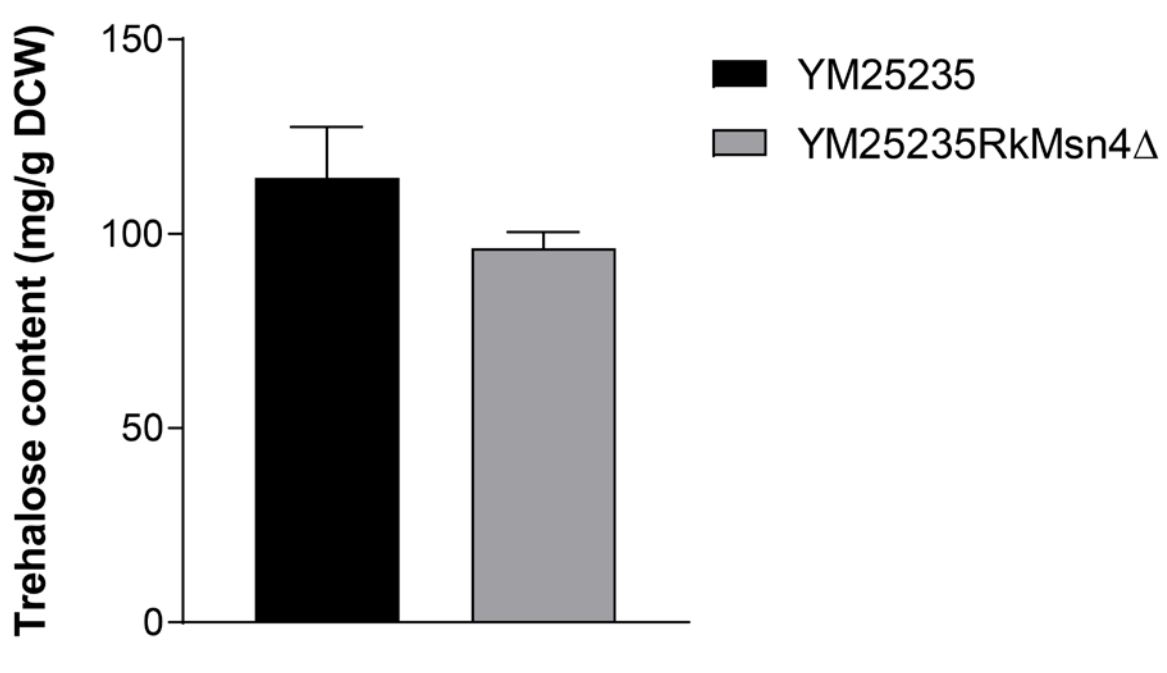

Fig. S9. Analysis of trehalose content under salt stress.

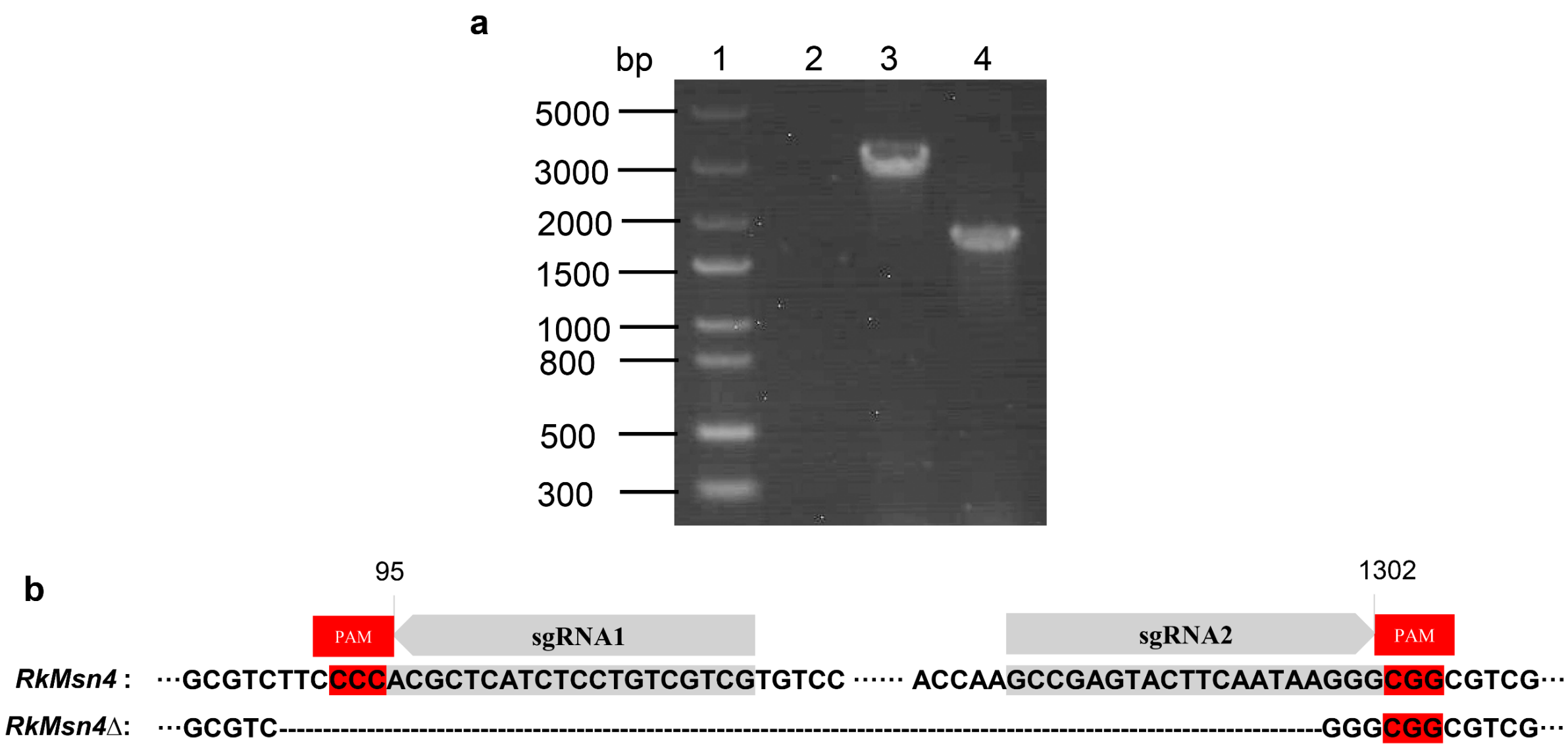

Fig. S10. PCR and sequencing validation of *RkMsn4* deletion mutant strain. **(a)** Agarose gel analysis for *RkMsn4* deletion mutant. 1. DNA marker; 2. Negative control; 3. PCR product of YM25235 amplified by the primers RkMsn4-F and RkMsn4-R; 4. PCR product of YM25235RkMsn4Δ was amplified with the primers RkMsn4-F and RkMsn4-R. **(b)** Sequencing of YM25235RkMsn4Δ. sgRNA sequence (gray), PAM sequence (red), deletions (black dashes).

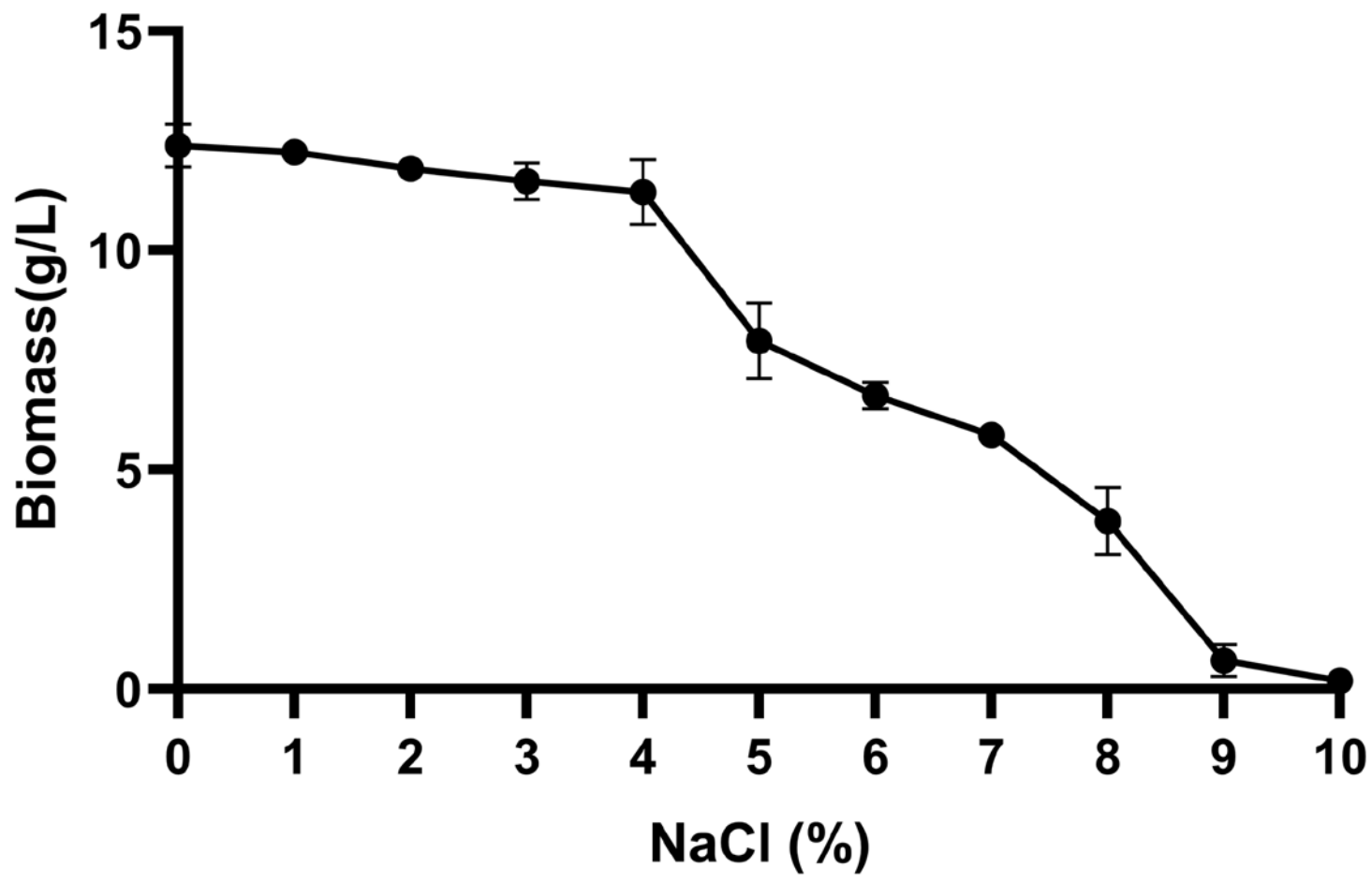

Fig. S11. Effect of different concentrations of NaCl on the growth of YM25235.
