# Supplementary material for: RkMsn4, a zinc finger transcription factor, regulates carotenoid and lipid metabolism to enhance tolerance to environmental stress in Rhodosporidium kratochvilovae
Source: Appl Environ Microbiol. 2026 Sep 23;92(10):e00647-26. doi: 10.1128/aem.00647-26 (PMC13689428; doi:10.1128/aem.00647-26)
Supplement: Supplementary file 2 [file aem.00647-26-s0002.pdf]

**Table S1.** Analysis of STRE motifs in promoters of genes related to antioxidant enzymes, carotenoid and lipid metabolism.

| Gene name | Gene_ID | $C_4T$_count | $C_4T$_position | $AG_4$_count | $AG_4$_position |
|---|---|---|---|---|---|
| *RkAcat2* | EVM0000476 | 4 | -1461 to -1457, -1436 to -1432, -500 to -496, -179 to -175 | 1 | -250 to -246 |
| *RkHMGCS* | EVM0001782 | 0 | | 0 | |
| *RkHMGCR* | EVM0000319 | 3 | -807 to -803, -133 to -129, -13 to -9 | 6 | -1876 to -1872, -1625 to -1621, -1503 to -1499, -1281 to -1277, -1222 to -1218, -866 to -862 |
| *RkMK* | EVM0004259 | 0 | | 0 | |
| *RkCrtYB* | EVM0000273 | 0 | | 0 | |
| *RkCrtI* | EVM0000700 | 2 | -974 to -970, -627 to -623 | 1 | -404 to -400 |
| *RkCTT1* | EVM0001925 | 3 | -1288 to -1284, -461 to -457, -129 to -125 | 5 | -1981 to -1977, -1847 to -1843, -1460 to -1456, -1018 to -1014, -994 to -990 |
| *RkSOD2* | EVM0005973 | 1 | -1104 to -1100 | 4 | -1834 to -1830, -1788 to -1784, -1373 to -1369, -45 to -41 |
| *RkGpx1* | EVM0006305 | 6 | -1803 to -1799, -1789 to -1785, -1504 to -1500, -1188 to -1184, -873 to -869, -52 to -48 | 1 | -1107 to -1103 |
| *RkGR* | EVM0006953 | 3 | -1812 to -1808, -1510 to -1506, -76 to -72 | 2 | -829 to -825, -461 to -457 |
| *RkACOX2* | EVM0004551 | 3 | -448 to -444, -362 to -358, -43 to -39 | 2 | -533 to -529, -262 to -258 |
| *RkACAA1* | EVM0006669 | 3 | -1912 to -1908, -1382 to -1378, -1092 to -1088 | 1 | -1595 to -1591 |
| *RkFAS1* | EVM0003722 | 8 | -1976 to -1972, -1202 to -1198, -1083 to -1079, -863 to -859, -487 to -483, -423 to -419, -62 to -58, -38 to -34 | 6 | -1012 to -1008, -683 to -679, -563 to -559, -236 to -232, -226 to -222, -130 to -126 |
| *RkACCS* | EVM0000452 | 0 | | 0 | |
| *RkOLE* | EVM0005779 | 2 | -839 to -835, -539 to -535 | 6 | -1980 to -1976, -1658 to -1654, -1113 to -1109, -1106 to -1102, -1101 to -1097, -615 to -611 |

**Table S2.** Statistics of transcriptome sequencing data.

| Stress condition | Sample | Raw reads | Clean reads | Clean bases | Q30(%) | Total mapped |
|---|---|---|---|---|---|---|
| Glucose starvation | YM25235_1 | 45,091,992 | 44,469,914 | 6,615,063,476 | 94.91 | 34,862,659 (78.40%) |
| | YM25235_2 | 45,697,846 | 45,054,360 | 6,721,376,780 | 94.79 | 36,351,121 (80.68%) |
| | YM25235_3 | 45,646,688 | 44,959,702 | 6,715,410,502 | 94.56 | 36,523,759 (81.24%) |
| | RkMsn4D_1 | 46,315,594 | 45,670,270 | 6,809,480,426 | 94.79 | 34,713,754 (76.01%) |
| | RkMsn4D_2 | 50,307,578 | 49,528,686 | 7,405,572,456 | 94.58 | 40,060,129 (80.88%) |
| | RkMsn4D_3 | 47,323,524 | 46,655,888 | 6,985,183,525 | 94.80 | 36,386,352 (77.99%) |
| Salt stress | YM25235_1 | 45,143,858 | 44,514,642 | 6,619,067,838 | 94.91 | 38,007,080 (85.38%) |
| | YM25235_2 | 41,054,698 | 40,481,914 | 6,030,593,890 | 94.88 | 34,088,614 (84.21%) |
| | YM25235_3 | 41,770,236 | 41,265,258 | 6,152,361,749 | 95.07 | 34,545,877 (83.72%) |
| | RkMsn4D_1 | 42,296,302 | 41,699,474 | 6,224,751,430 | 94.90 | 34,757,585 (83.35%) |
| | RkMsn4D_2 | 44,581,764 | 43,946,840 | 6,555,010,935 | 94.87 | 36,562,264 (83.20%) |
| | RkMsn4D_3 | 45,622,424 | 45,050,896 | 6,713,529,052 | 95.07 | 37,670,318 (83.62%) |
| Low temperature | Y235_15_1 | 45,296,520 | 44,779,040 | 6,716,621,127 | 94.40 | 37,953,295 (84.76%) |
| | Y235_15_2 | 45,525,124 | 45,041,010 | 6,764,817,502 | 94.81 | 37,816,387 (83.96%) |
| | Y235_15_3 | 41,504,890 | 41,131,648 | 6,177,303,169 | 94.44 | 34,769,970 (84.53%) |
| | RkMsn4D_1 | 39,670,974 | 39,303,096 | 5,901,607,489 | 94.50 | 33,131,782 (84.30%) |
| | RkMsn4D_2 | 44,590,116 | 44,082,230 | 6,617,119,067 | 94.40 | 37,012,801 (83.96%) |
| | RkMsn4D_3 | 44,067,012 | 43,640,158 | 6,553,618,813 | 94.47 | 36,834,011 (84.40%) |

**Table S4.** Key genes in different modules.

| Module | Gene ID | Gene Description | $C_4T$_count | $AG_4$_count |
|---|---|---|---|---|
| MEbrown | EVM0003520 | ------ | 0 | 0 |
| | EVM0002336 | CAMK/CAMKL/PASK protein kinase | 4 | 2 |
| | EVM0005349 | C2H2-type zinc finger | 0 | 2 |
| | EVM0005444 | bZIP transcription factor | 2 | 15 |
| | EVM0003387 | ------ | 11 | 1 |
| | EVM0007429 | BZIP-type transcription factor MBZ1 | 2 | 10 |
| | EVM0000551 | Reverse transcriptase | 6 | 7 |
| | EVM0003316 | GDP-fucose protein O-fucosyltransferase | 1 | 2 |
| | EVM0001177 | MFS-type transporter MFS54 | 0 | 0 |
| MEpink | EVM0000512 | hypothetical protein | 1 | 2 |
| | EVM0002216 | hypothetical protein | 2 | 0 |
| MEgreen | EVM0003067 | Zinc-regulated transporter 1 | 0 | 1 |
| MEblack | EVM0004841 | Phosphatidate phosphatase | 2 | 3 |
| | EVM0000716 | ------ | 0 | 0 |
| | EVM0003539 | PHO85 cyclin-1 | 3 | 2 |
| | EVM0007330 | 2-oxoacid dehydrogenases acyltransferase-domain-containing | 3 | 2 |

| | | | | |
|---|---|---|---|---|
| | | protein | | |
| | EVM0005382 | Putative MFS transporter | 3 | 0 |
| | EVM0005344 | 2-oxoacid dehydrogenases acyltransferase-domain-containing protein | 0 | 0 |
| MEblue | EVM0002663 | Septin ring organizing protein mid2 | 3 | 1 |
| | EVM0000051 | ATP adenylyltransferase C-terminal domain | 0 | 0 |
| | EVM0001912 | Serine/threonine-protein kinase sid2 | 4 | 5 |
| | EVM0001948 | ATP-dependent DNA helicase PIF1 | 3 | 5 |
| | EVM0005157 | ------ | 0 | 0 |
| | EVM0005491 | Structural maintenance of chromosomes protein 4 | 3 | 1 |
| | EVM0007891 | ------ | 2 | 8 |
| MEturquoise | EVM0001410 | Mpv17 / PMP22 family | 0 | 4 |
| | EVM0004607 | dimethylaniline monooxygenase | 4 | 1 |
| | EVM0003649 | Major facilitator superfamily domain-containing protein | 0 | 2 |

**Table S5.** sgRNA design table.

| | 20-nt gRNA (NGG) (5'-3') | gRNA position |
|---|---|---|
| SgRNA1 | CGACGACAGGAGATGAGCGT(GGG) | -114 to -95 |
| SgRNA2 | GCCGAGTACTTCAATAAGGG(CGG) | 1283 to 1302 |

**Table S6.** *RkMsn4* full-length primers for PCR validation.

| | Primer sequence (5'-3') |
|---|---|
| RkMsn4-F | ATGGCGCCTGCTCCCCGTGGT |
| RkMsn4-R | TCACGGCATCGCCCACACC |

**Table S7.** Primer pairs used for quantitative real-time PCR.

| Gene | Primer sequence (5'-3') | |
|---|---|---|
| *SSU rRNAs* | F: CCATTCACTTACAAACACAA | R: CACCACCAGATTCACTAA |
| *RkAcaT2* | F: CCGAACATTATGGGCATC | R: CGATGTCCTCAATCTTCAT |
| *RkHMGCS* | F: TTCCTCCTCTCCGTGACCAA | R: CGTCTTGACCGACTTGCTCT |
| *RkHMGCR* | F: CATTGTCACCGTCTTCTG | R: GTCGTCGTAGAGGAGGAA |
| *RkMK* | F: ACACGATCAACCCGCTCTTC | R: GAGGGACGAAAGCTCAACGA |
| *RkCrtYB* | F: GTTCTTCTTGTGGGAGTG | R: CCGCTTCTTCAATCTCAA |
| *RkCrtI* | F: GAGAAGGGTTTCGAGGGCTT | R: ATGGAGAGGAGCGAGGTGAA |
| *RkACOX2* | F: ATTCACGACCTCACCAAGGC | R: CACTCCATATCGCGTCCAGA |
| *RkACAA1* | F: GTCGAGTCGATGACCCAAGG | R: ACGTTCTCGCTCGTGATACC |
| *RkFAS1* | F: ACACTACAACGAGAAGGCCG | R: CGCTGGTTGATGTAGAGGCA |

| | | |
|---|---|---|
| *RkACCS* | F: GCACGACCGAGTTCCTCTAC | R: GTTGACGCCCGAAACCATTT |
| *RkCTT1* | F: CAGGTCATCCCCGACAACTC | R: GAGGGTCTTTGAGCGGTTGA |
| *RkSOD2* | F: CGCATGTCCTGACTCTCTCC | R: AGGTTCTCCCAGAAGAGCGA |
| *RkGpx1* | F: GAGAAGCCTAACGGCACCAA | R: GAACTGGTCCGACGGGAAG |
| *RkGR* | F: TTCTACGCGATGATGGAGCC | R: CAAAGCCCTGGAGCATCTCA |
